# Teaching Pain Management in Serious Illness in the Era of the Opioid Epidemic: A Team-Based Intervention

**DOI:** 10.15766/mep_2374-8265.11006

**Published:** 2020-10-30

**Authors:** Alana Sagin, Sharon M. Kimberly, Jill P. Farabelli, Kava Schafer, Pallavi Kumar, Tanya J. Uritsky

**Affiliations:** 1 Assistant Professor, Palliative Care, University of Pennsylvania's Perelman School of Medicine; 2 Licensed Clinical Social Worker, Palliative Care, Hospital of the University of Pennsylvania; 3 Chaplain, Palliative Care, Hospital of the University of Pennsylvania; 4 Assistant Professor, Hematology Oncology and Palliative Care, University of Pennsylvania's Perelman School of Medicine; 5 Clinical Pharmacy Specialist, Pain Medication Stewardship, Hospital of the University of Pennsylvania

**Keywords:** Palliative Care, Opioid Risk Mitigation, Pain Management, Interdisciplinary Team, Opioids

## Abstract

**Introduction:**

Despite the prevalence of pain in patients with serious illness, recent guidelines for opioid prescribing practices have largely excluded palliative care patients. In lieu of such guidelines, many have recommended adapting risk mitigation strategies from the chronic pain arena for palliative care and oncology populations. Teaching interventions are needed to demonstrate how these methods can be applied to patients with serious illness.

**Methods:**

We developed a teaching intervention for fourth-year medical students to improve knowledge about safe opioid prescribing practices in palliative care patients and emphasized both effective and safe pain management. A secondary aim of the intervention was to demonstrate how a palliative care interdisciplinary team works together to care for a complex patient near the end of life. The intervention lasted 1 hour and consisted of an interdisciplinary case presentation as well as a slide presentation.

**Results:**

Twenty-two medical students attended the session over 2 years. After the intervention, medical students better understood risk mitigation strategies and felt more strongly that opioids can be a useful tool in treating pain for patients with serious illness. Students' familiarity with palliative care interdisciplinary roles also improved after the intervention.

**Discussion:**

This session was a useful part of a palliative care 2-week classroom elective and was well received by students. The development of a survey tool that assesses student attitudes around effective and safe pain management in patients with serious illness may be of use to others who teach pain management in palliative care populations.

## Educational Objectives

By the end of this activity, learners will be able to:

1. Define the concepts of total pain, pseudo-addiction, chemical coping, opioid use disorder (OUD), and diversion.
2. Identify challenges in caring for patients with OUD near the end of life.
3. Describe a four-step opioid risk mitigation approach for palliative care patients.
4. Describe how to safely taper an opioid regimen when appropriate.
5. Describe how a palliative care team works together to care for a patient with OUD and serious illness.

## Introduction

In the era of the opioid epidemic, teaching pain management in serious illness can be a challenge. While medical schools may be attempting to incorporate pain management and opioid risk mitigation education into their curricula, there are ongoing challenges such as limited time and inadequate faculty training.^1^ A recent literature review identified a lack of published teaching interventions for medical students on the topic of opioid use disorder (OUD) and concluded that more education in this area is needed.^2^

Palliative care is specialized care for people with serious illness, which has been defined as, “a health condition that carries a high risk of mortality and either negatively impacts a person's daily function or quality of life, or excessively strains their caregivers.”^3^ Pain in palliative care patients presents unique challenges in that many patients experience pain that is influenced by their serious illness or prognosis and the financial, psychosocial, and existential stressors that come with such a diagnosis. Recent guidelines for safe prescribing practices have largely excluded palliative care populations^4^ and there is a general lack of evidence for appropriate risk mitigation measures in this population. In lieu of such guidelines, many have recommended using a universal precautions approach in the palliative care and oncology populations, routinely screening for risk for OUD and using risk mitigation tools from the chronic pain arena.^5,6^

We designed our teaching intervention to be part of a 2-week classroom elective course on palliative care for fourth-year medical students at the University of Pennsylvania's Perelman School of Medicine. We have found through teaching pain management in the palliative care population that medical students may be worried about contributing to an OUD or be hesitant to learn about opioids at all if we fail to adequately discuss risk mitigation with them. Furthermore, in the setting of the current opioid crisis there is a need for further training on this topic which in the past has been underrepresented in medical school curricula.^7^ Recent *MedEdPORTAL* publications described interventions to teach safe prescribing practices in chronic pain to medical students.^8–10^ These interventions have been found to be useful; however, they have not addressed pain in serious illness directly. In an effort to improve medical student knowledge about safe prescribing practices and to impress upon them the importance of both safe and effective pain management as well as interdisciplinary team collaboration, we created a two-part teaching intervention consisting of an interdisciplinary case presentation and a slide presentation.

## Methods

We gave the intervention in the fall of 2018 and again in the fall of 2019. We intentionally gave this intervention before the bulk of the sessions on pain management as we felt it was important to address students' anxieties about the potential for OUD before we could effectively teach the intricacies of pain management. The session was presented in a small-group format. The first year the intervention was 2 hours long and consisted of three parts: a case presentation, a didactic presentation on opioid prescribing and risk mitigation strategies, and a panel discussion. The second year, based on student feedback, we shortened the intervention to 1 hour and removed the panel discussion. We also decreased the number of presenters from six to four.

The first part of the intervention involved a 30-minute multidisciplinary case presentation of a patient nearing the end of life with increased risk for OUD (Appendix A). Presenters in this teaching intervention included a social worker, spiritual care provider, and a pharmacist from the University of Pennsylvania Health System's palliative care consult service, as well as a physician who works in the palliative care outpatient clinic. Presenters took turns describing parts of the case where they interacted with the patient to demonstrate how members of the multidisciplinary team work together. A moderator for the session was also present to facilitate introductions, transitions, and timekeeping.

The case described a 32-year-old man with advanced acute lymphocytic leukemia, chronic anxiety, and cancer pain, as well as chronic noncancer pain. He had psychosocial and financial stressors as well as existential distress. The case followed him from the inpatient setting where the palliative care pharmacist, spiritual care provider, and social worker were involved in his care, to the outpatient palliative care clinic and back to the inpatient setting where he eventually transitioned to hospice care. Throughout his care there were multiple aberrant drug-related behaviors around his opioid use and he was felt to be at increased risk for OUD. The team responded in multiple ways including increasing his visit frequency, increasing psychosocial support, and decreasing his opioids at times. Presenters also spoke about bias in the healthcare system and their own emotional responses to this complex situation.

The second part was a 30-minute PowerPoint presentation on opioid risk assessment and management in palliative care (Appendix B). It discussed a four-part approach adapted from the Center to Advance Palliative Care's Pain Management Modules that included eliciting a substance use history, screening using a risk assessment tool, developing a management plan based on risk, and monitoring for at-risk behavior.^11^ This was given in segments between pauses in the clinical case presentation and was presented by a palliative care pharmacist.

### Evaluation and Analysis

We obtained institutional review board exemption for the analysis of our intervention. We designed a 13-question Likert survey intended to assess self-perceived familiarity toward opioid use and interdisciplinary teams in palliative care. In our review of the literature we found that existing surveys evaluating attitudes around opioid prescribing either predated the current opioid crisis and minimized the possibility of adverse effects from opioids,^12^ or were more current but focused on chronic pain and the importance of avoiding opioids.^13^ We obtained feedback from other palliative care providers at our institution until we were satisfied with a final iteration (Appendix C). Our students completed the survey immediately before and after the teaching interventions, as well as approximately 3 months after the interventions. Results were anonymous. We also elicited feedback about the intervention in a free-form question immediately after the interventions.

## Results

In 2018, eight students attended the teaching session, and in 2019 14 students attended. All 22 students who attended the sessions responded to the surveys immediately before and after the intervention. Despite the changes made between the first and the second years, most notably cutting the session time in half, we did not note any major differences in survey scores between the first and second years. As detailed in the Table, survey responses demonstrated that students were more comfortable with opioid risk mitigation strategies after the interventions. In response to the statement “I am familiar with some strategies to help guide me when caring for patients with high risk of substance use disorder and pain in the setting of serious illness” the average student response on a 5-point Likert scale (1 = *strongly disagree*, 5 = *strongly agree*) immediately after was higher (*M* = 3.9) than immediately before (*M* = 2.5).

**Table.**
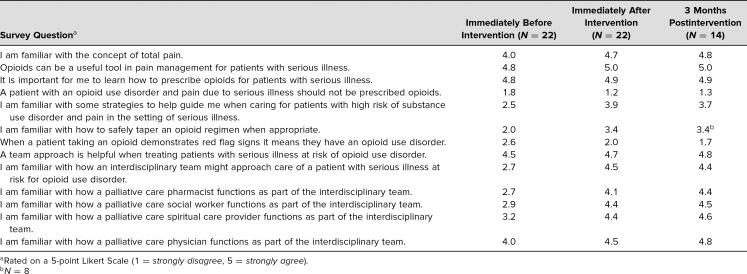
Average Survey Scores Immediately Before, Immediately After, and 3 Months After Educational Intervention (2018 and 2019)^a^

Students' familiarity with the palliative care team and individual roles also improved. While only one of the students strongly agreed that they were familiar with how an interdisciplinary team might approach care of a patient with serious illness at risk for OUD before the intervention (*M* = 2.7), most of them strongly agreed after the intervention (*M* = 4.5), and they felt more strongly that a team approach is helpful (*M*_before_ = 4.5, *M*_after_ = 4.7). Similarly, after the intervention students better understood the concept of total pain (*M*_before_ = 4.0, *M*_after_ = 4.7) and felt more strongly that opioids can be a useful tool in treating pain for patients with serious illness (*M*_before_ = 4.8, *M*_after_ = 5.0).

Not all students responded to the follow-up survey at 3 months (*N* = 14) and one survey question regarding opioid tapering was unintentionally left off of a follow-up questionnaire in the 2018 cohort. However, the results for all questions showed sustained improvements in knowledge and attitudes (Table). We also collected feedback from students both verbally and anonymously in a free form survey question. Most felt positive about the session and found it helpful. We did receive feedback that 2 hours felt long after the first year and we changed the session to be only 1 hour the second year. Both years we received feedback that the session could be more interactive.

## Discussion

This session was a useful part of our palliative care 2-week classroom elective for medical students. The session was well received by the students, and our findings showed that the intervention improved student's self-perceived comfort with pain management and risk mitigation strategies in patients in serious illness, as well as interdisciplinary roles on the palliative care team. This adds to the literature in this area in that it specifically addresses pain management in serious illness, an underaddressed topic.

Our teaching intervention for pain management in palliative care may be useful to others in the oncology or palliative care realms, and could be relevant to learners of various disciplines and at various stages in their curricula. The survey to evaluate knowledge and attitudes of medical students around pain management and risk mitigation may be of particular interest to other providers of medical education seeking to emphasize both safe and effective opioid prescribing practices in serious illness.

There were several limitations that may affect the generalizability of our results. First, there were only 22 students who enrolled in the session and completed our survey, and only 14 completed the follow-up survey at 3 months. Secondly, there was selection bias as the students evaluated had signed up for the elective in palliative care and thus were interested in palliative care already. Third, although our assessment tool was reviewed for content by multiple palliative care specialists, it was not rigorously validated. Finally, the lack of skills assessment and the relatively passive approach to learning was a limitation that was apparent in the feedback we received from students.

Challenges to implementation of this session included the depth of such a large topic in a limited amount of time, lack of strong evidence around the topics discussed, and the availability of small conference rooms where such a session is best suited. An example of the depth of the topic that presented a challenge was the difference in recommendations regarding urine drug screen monitoring in the chronic pain population where global screening is recommended, and in the palliative care population where guidelines recommend urine drug screening for patients in an increased risk group.^11^ While we touched on these differences in our session, a full discussion of how we treat patients differently based on pain category is one that was difficult given limited time and lack of literature around this topic.

We plan to continue to include this session in the palliative care classroom elective course that meets each October. We will also assess the feasibility of incorporating the intervention into the general medical student curricula. Based on student feedback we will develop ways to make the session more interactive and develop take-home materials. In the future we will also assess the feasibility of further statistical analysis of our results and further development of our survey tool.

## Appendices

Case.docxPain Management & Risk Presentation.pptxPain & Risk Survey.docx
All appendices are peer reviewed as integral parts of the Original Publication.
