# Supplementary material for: Teaching Pain Management in Serious Illness in the Era of the Opioid Epidemic: A Team-Based Intervention
Source: MedEdPORTAL. 2020 Oct 30;16:11006. doi: 10.15766/mep_2374-8265.11006 (PMC7597940; doi:10.15766/mep_2374-8265.11006)
Supplement: Supplementary file 1 — Case.docxPain Management & Risk Presentation.pptxPain & Risk Survey.docx [file mep_2374-8265.11006-s001.zip › A. Case.docx]

**Appendix A: Summary of Multidisciplinary Case Presentation**

The case is presented in a small group setting, chairs should ideally be arranged in a circle to facilitate interaction with learners. The case is presented by the interdisciplinary team and although each team member may not have a large amount of speaking time their presence deepens the experience of the learner as they get first-hand perspective of the role of each individual contributor to the team. There is also a moderator for the session to facilitate introductions, transitions and time-keeping.

**Part 1:**

**Presented by pharmacist:**

B is a 32 year old man with relapsed acute lymphocytic leukemia, chronic anxiety and chronic pain admitted to the hospital for chemotherapy. He had been taking oxycodone for his pain for many years but has no history of substance use disorder. Family history includes a father with an alcohol use disorder. B’s team has continued his home pain regimen of oxycodone ER, oxycodone IR PRN and gabapentin. The team is worried because of perceived contradictions between what he says is his regimen and outpatient notes from pain management. His pain is poorly controlled. Palliative Care is consulted to help with pain management in the setting of advanced illness.

The palliative care pharmacist evaluates him and checks the Prescription Drug Monitoring Program (PDMP) however data is not available as he lives out of state and multi-state PDMP is not available. Due to his family history he has increased risk for opioid use disorder but no aberrant drug related behaviors. She feels his pain is due to progression of disease and recommends a hydromorphone PCA and steroids. He is converted to methadone and pregabalin and discharged with well-controlled pain and improved function.

**Presented by social worker:**

During this hospitalization the palliative care social worker provides support for B’s wife as B is often in pain and not up for talking. They are small business owners and recently had to close the business as B has been more recently unable to work. He has two young children 12 and 4 years old, the youngest has a developmental disability. Outside of the room B’s wife often reveals her own struggles with anxiety, depression and past abusive relationships. Pain management has been very difficult for B and is a topic they discuss often and with much distress. Wife expresses some concerns that he sometimes seems too reliant on the pain medication.

**Presented by chaplain:**

The palliative care spiritual counselor meets with B and finds out that he is of a Christian faith background. He shares that he was molested by a priest as a child. He has never spoken of this before to anyone. This abuse connected to his religiosity and he has turned away from the church. He wonders why God did not protect him.

**Part 2:**

**Presented by physician:**

B follows up in palliative care clinic and sees a palliative care physician and social worker for pain management and support. Initially his methadone is increased but his pain and functioning do not seem to improve with the increase. He also shows some aberrant drug related behaviors such as missed appointments and chemical coping. A strict monitoring and treatment plan is enforced and a plan made to decrease his opioids back to previous doses. Social work continues to provide support. Goals of care are addressed and he wants to pursue all possible treatment options.

**Part 3**

**Presented by pharmacist:**

B is admitted from outpatient with severe uncontrolled pain. Scans reveal progression of disease. He is again seen by the inpatient palliative care team and started on a hydromorphone PCA. His outpatient oncologist recommends hospice.

**Presented by social worker:**

The palliative care social worker met with B’s wife and 12 year old several times and discussed legacy projects, assessed bereavement needs and coordinated with on-site hospice liaison. There are multiple goals of care conversations after which B transitions to inpatient hospice and dies shortly after in the hospital.

**Talking Points/Questions:**

1. How might stigma and stigmatizing language in the EMR have affected his care?

*Stigmatizing language in the EMR can affect clinician attitudes toward the patient and subsequent medical decision making. For example, clinicians may be less likely to increase pain medication.*

1. What do you need to or want to know about his psychosocial and financial situation? His history of abuse?

*Understanding more about his life stressors is important to understand his current psychological struggles and coping mechanisms as well as shed light on dynamics between family members. This information may also reveal unmet needs that could be addressed by the medical team.*

1. Explain how different team members added to his care?

*This patient is suffering from multiple forms of distress. He experiences existential distress that can best be addressed by pastoral care. His wife struggles to help with his healthcare as well as raise their young children and they have financial stressors. Social work may be best suited to provide support and resources around these stressors. The palliative care pharmacist and physician are involved in managing his pain. These different roles serve to address the patient’s suffering from multiple angles.*

1. Describe how this case is different from non-malignant pain being managed in a primary care office? How is it similar?

*Similarities include the need for opioid risk mitigation practices, a multimodal approach to pain management and a wholistic support model. A difference is the added source of suffering caused by the patient’s poor prognosis and resulting psychosocial and existential distress which are undoubtedly complicating his experience of physical pain. This is a good opportunity to point out the ethical dilemma faced by clinicians when patient are at high risk for opioid use disorder or have an opioid use disorder and at the same time have severe pain at the end of life.*
