# Supplementary material for: Teaching Pain Management in Serious Illness in the Era of the Opioid Epidemic: A Team-Based Intervention
Source: MedEdPORTAL. 2020 Oct 30;16:11006. doi: 10.15766/mep_2374-8265.11006 (PMC7597940; doi:10.15766/mep_2374-8265.11006)
Supplement: Supplementary file 1 — Case.docxPain Management & Risk Presentation.pptxPain & Risk Survey.docx [file mep_2374-8265.11006-s001.zip › B. Pain Management & Risk Presentation.pptx]

## Slide 1
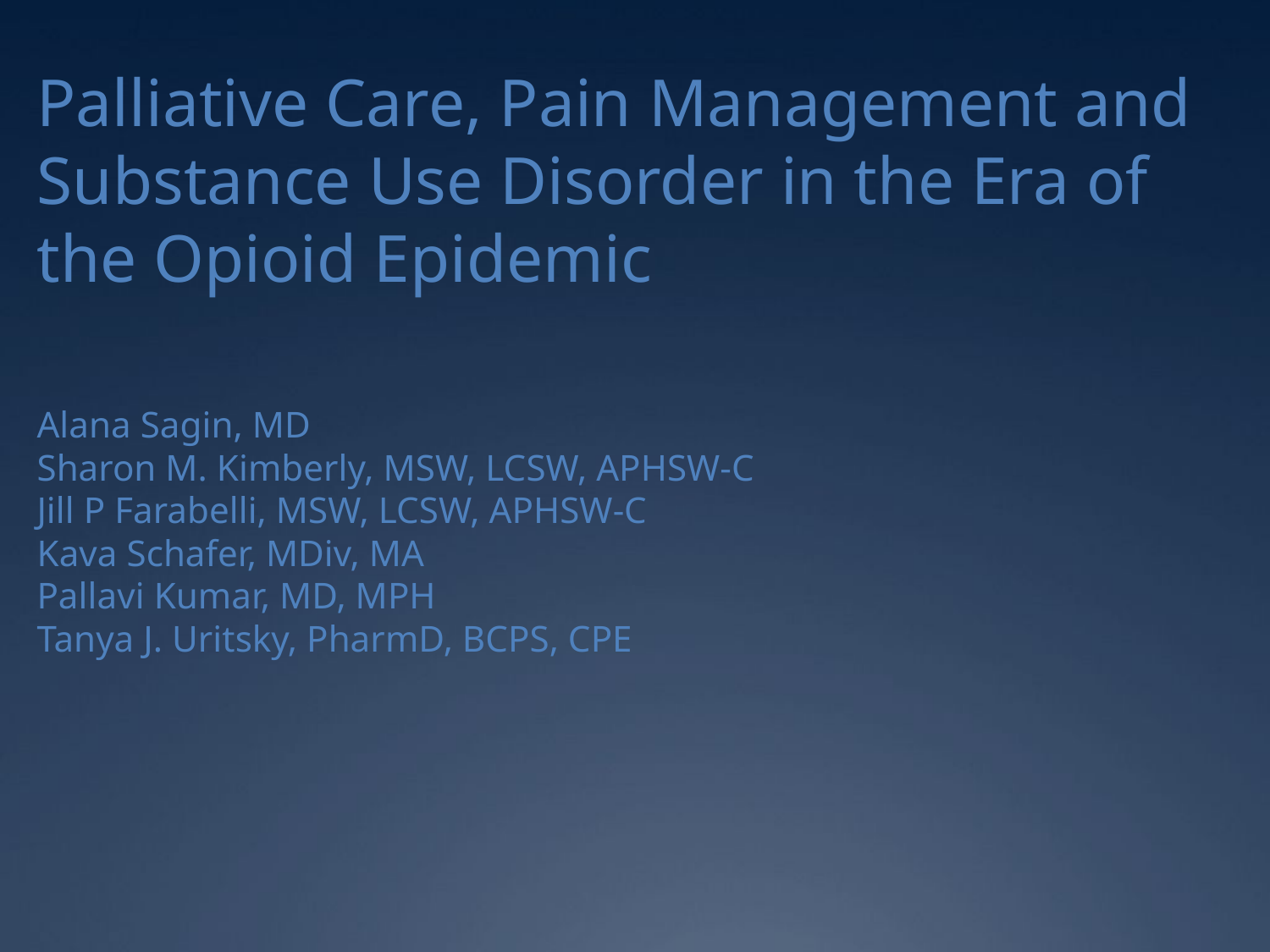

Palliative Care, Pain Management and Substance Use Disorder in the Era of the Opioid Epidemic
Alana Sagin, MD
Sharon M. Kimberly, MSW, LCSW, APHSW-C
Jill P Farabelli, MSW, LCSW, APHSW-C
Kava Schafer, MDiv, MA
Pallavi Kumar, MD, MPH
Tanya J. Uritsky, PharmD, BCPS, CPE
#

## Slide 2
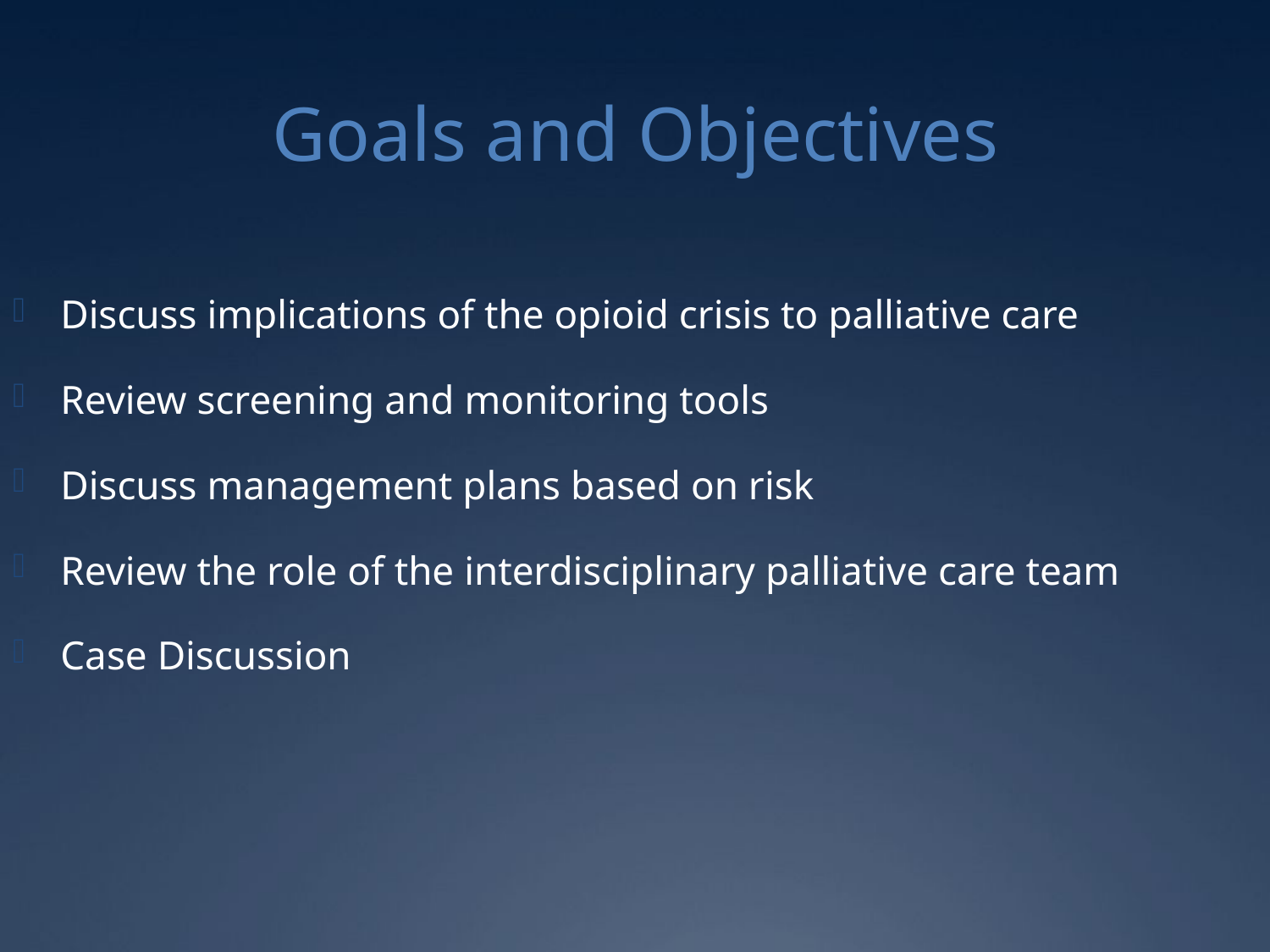

# Goals and Objectives
Discuss implications of the opioid crisis to palliative care
Review screening and monitoring tools
Discuss management plans based on risk
Review the role of the interdisciplinary palliative care team
Case Discussion

## Slide 3
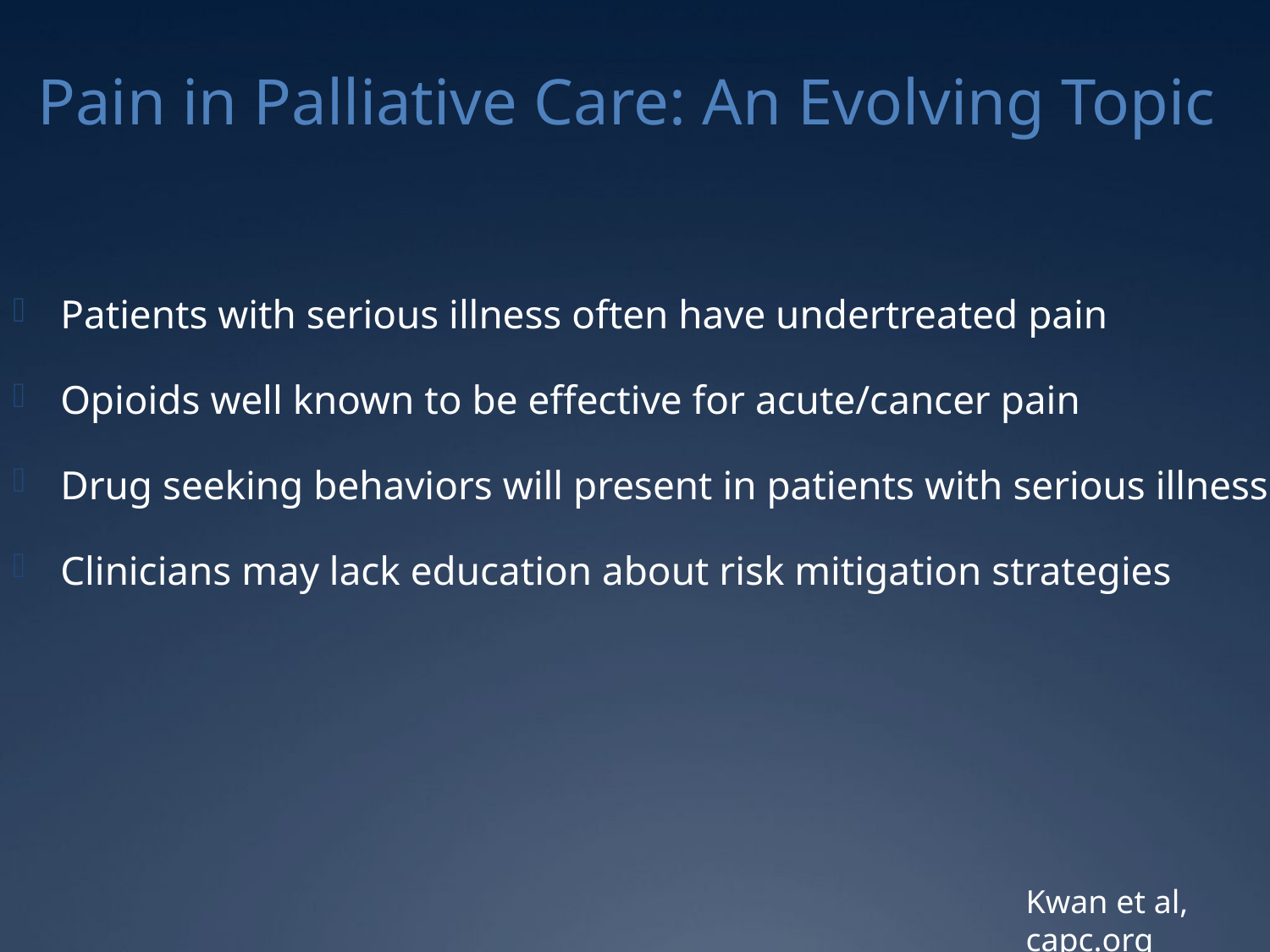

# Pain in Palliative Care: An Evolving Topic
Patients with serious illness often have undertreated pain
Opioids well known to be effective for acute/cancer pain
Drug seeking behaviors will present in patients with serious illness
Clinicians may lack education about risk mitigation strategies
Kwan et al, capc.org

## Slide 4
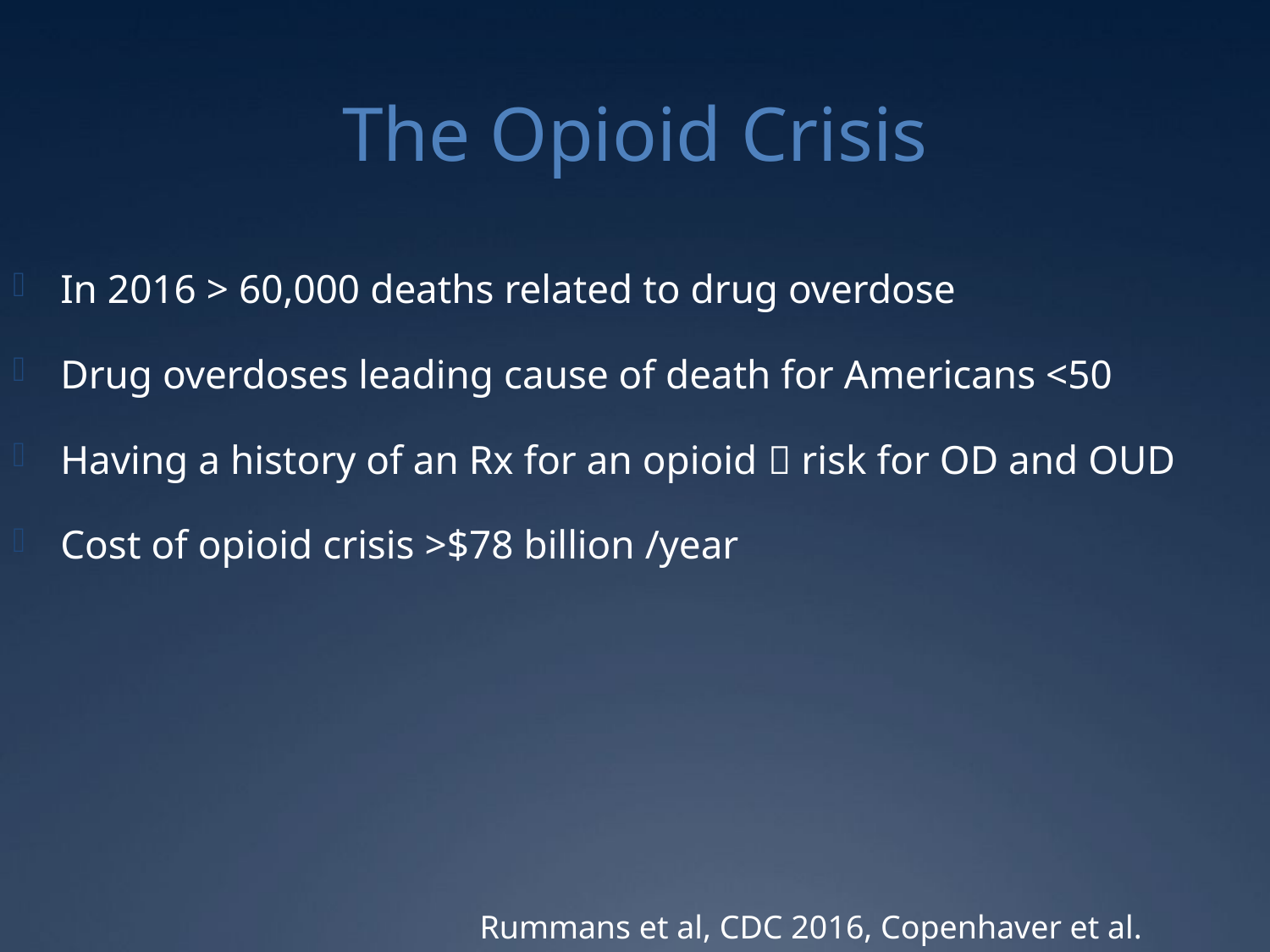

# The Opioid Crisis
In 2016 > 60,000 deaths related to drug overdose
Drug overdoses leading cause of death for Americans <50
Having a history of an Rx for an opioid  risk for OD and OUD
Cost of opioid crisis >$78 billion /year
Rummans et al, CDC 2016, Copenhaver et al.

## Slide 5
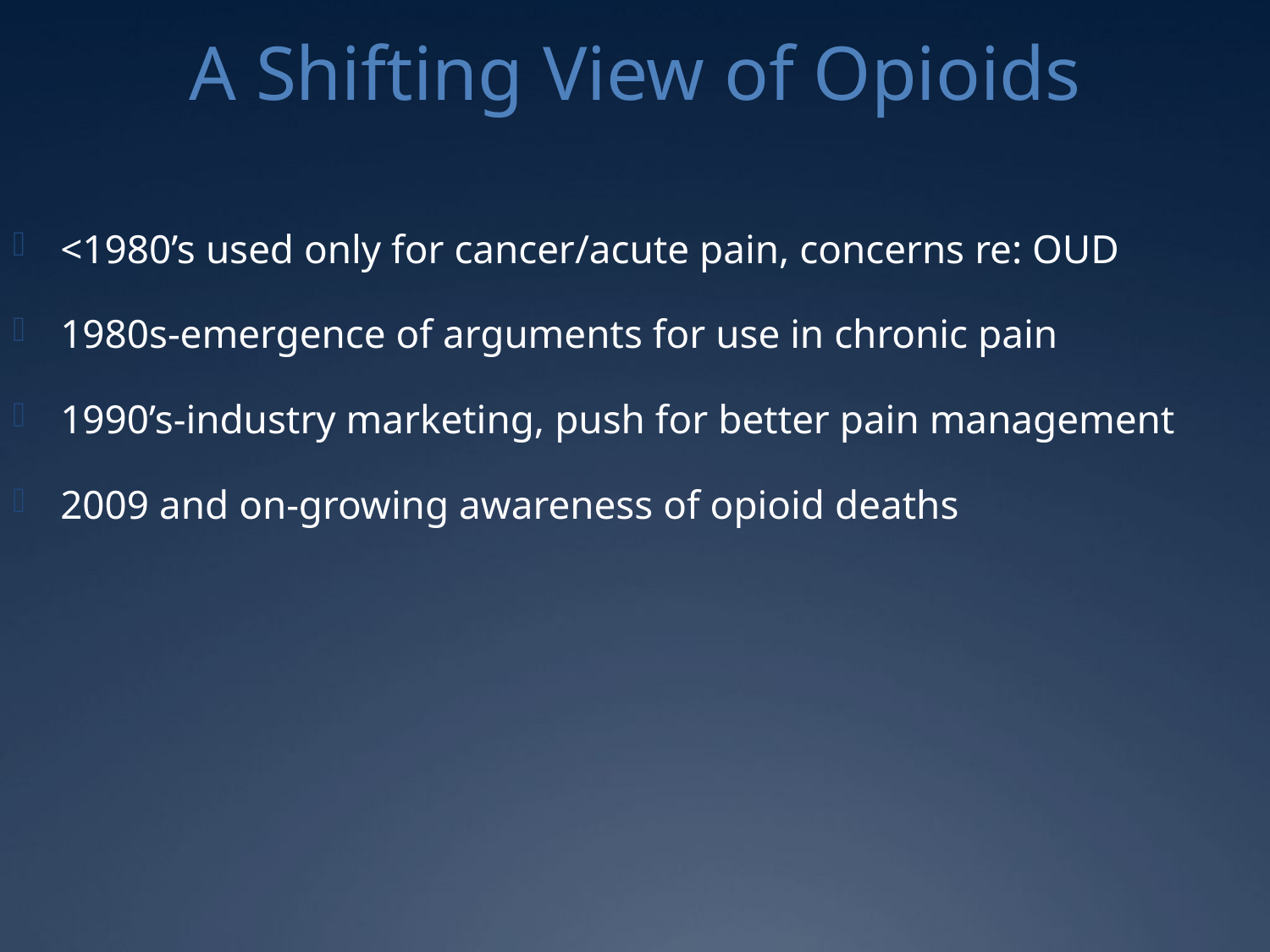

# A Shifting View of Opioids
<1980’s used only for cancer/acute pain, concerns re: OUD
1980s-emergence of arguments for use in chronic pain
1990’s-industry marketing, push for better pain management
2009 and on-growing awareness of opioid deaths

## Slide 6
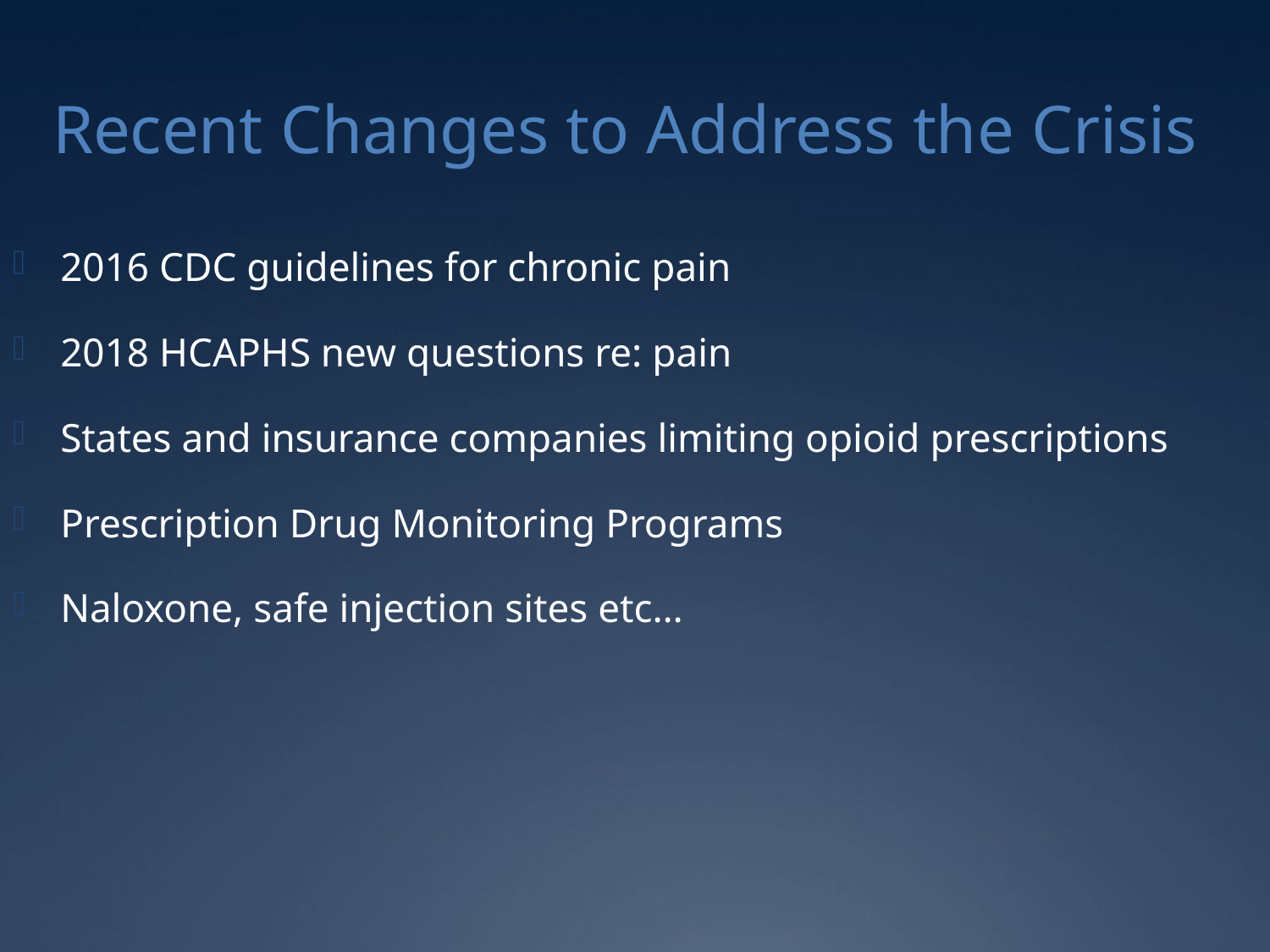

# Recent Changes to Address the Crisis
2016 CDC guidelines for chronic pain
2018 HCAPHS new questions re: pain
States and insurance companies limiting opioid prescriptions
Prescription Drug Monitoring Programs
Naloxone, safe injection sites etc…

## Slide 7
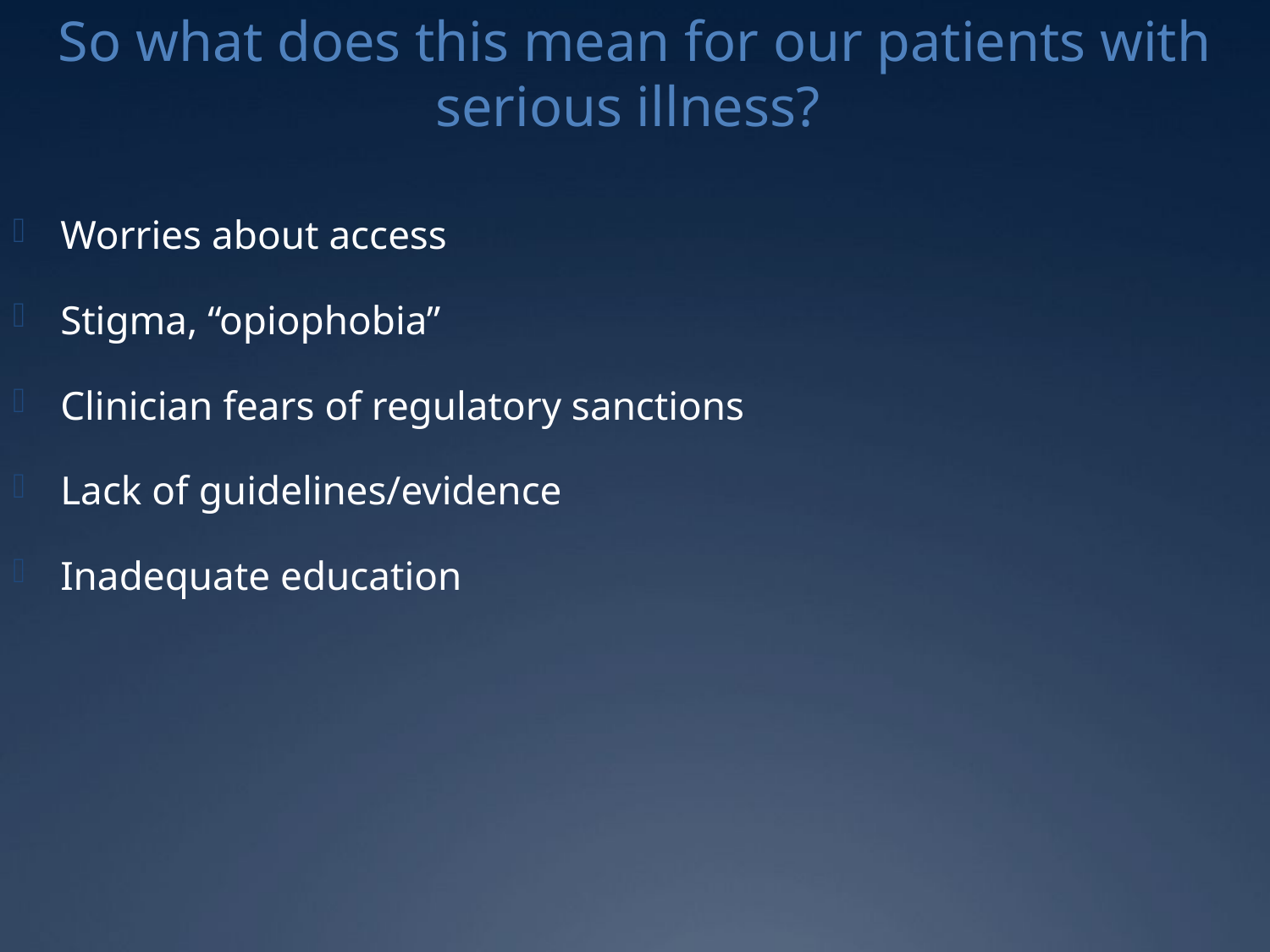

# So what does this mean for our patients with serious illness?
Worries about access
Stigma, “opiophobia”
Clinician fears of regulatory sanctions
Lack of guidelines/evidence
Inadequate education

## Slide 8
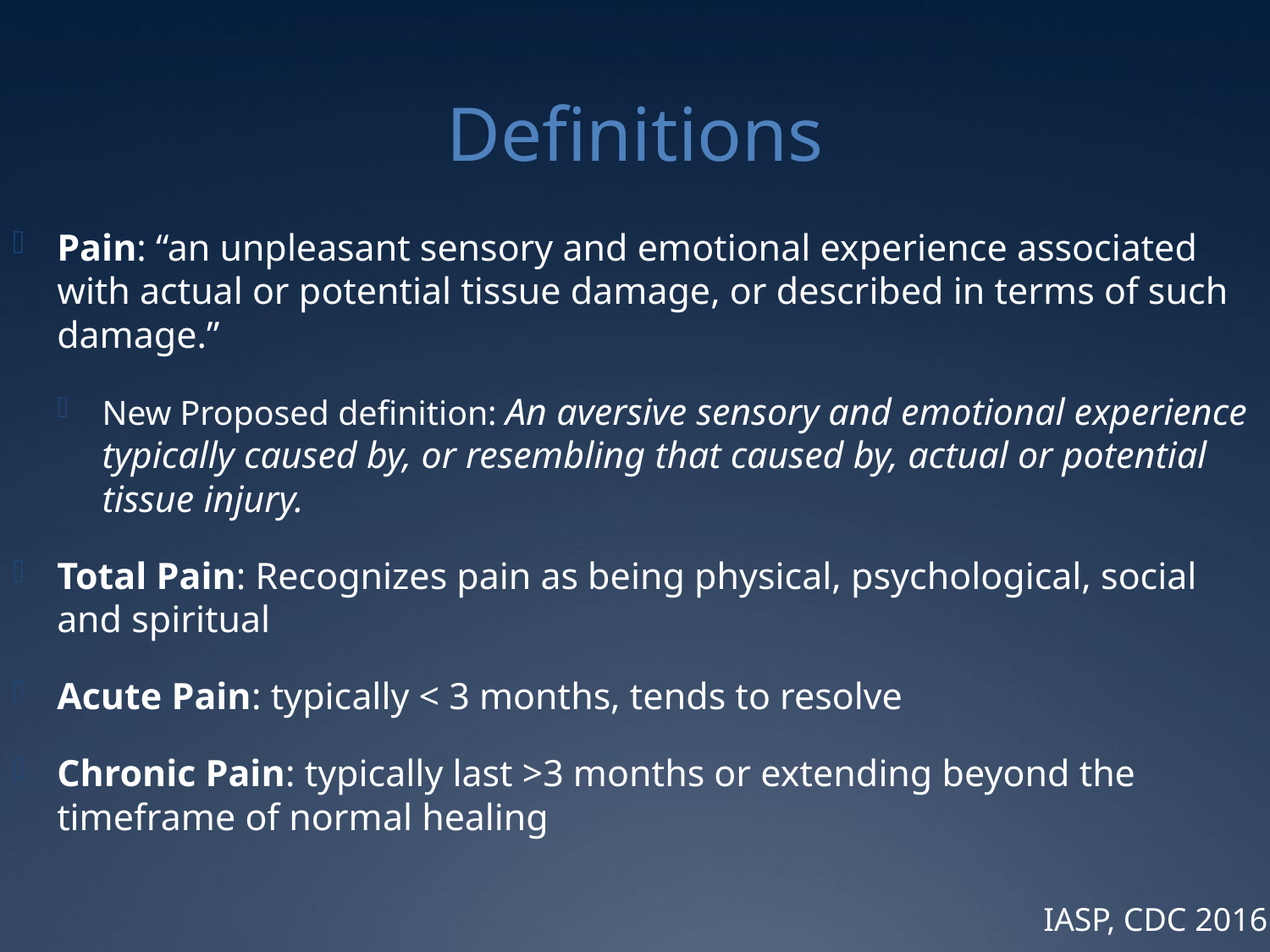

# Definitions
Pain: “an unpleasant sensory and emotional experience associated with actual or potential tissue damage, or described in terms of such damage.”
New Proposed definition: An aversive sensory and emotional experience typically caused by, or resembling that caused by, actual or potential tissue injury.
Total Pain: Recognizes pain as being physical, psychological, social and spiritual
Acute Pain: typically < 3 months, tends to resolve
Chronic Pain: typically last >3 months or extending beyond the timeframe of normal healing
IASP, CDC 2016

## Slide 9
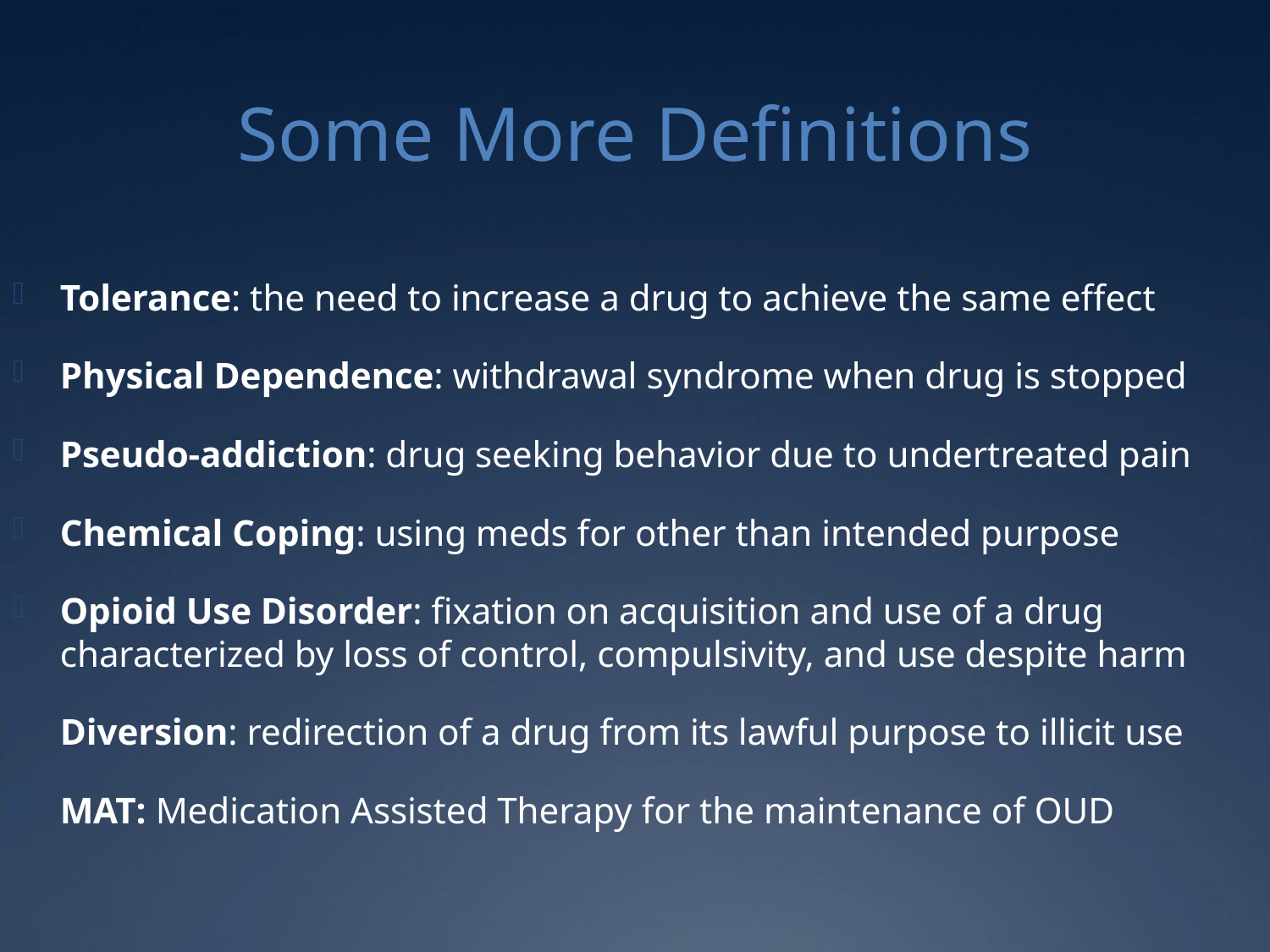

# Some More Definitions
Tolerance: the need to increase a drug to achieve the same effect
Physical Dependence: withdrawal syndrome when drug is stopped
Pseudo-addiction: drug seeking behavior due to undertreated pain
Chemical Coping: using meds for other than intended purpose
Opioid Use Disorder: fixation on acquisition and use of a drug characterized by loss of control, compulsivity, and use despite harm
Diversion: redirection of a drug from its lawful purpose to illicit use
MAT: Medication Assisted Therapy for the maintenance of OUD

## Slide 10
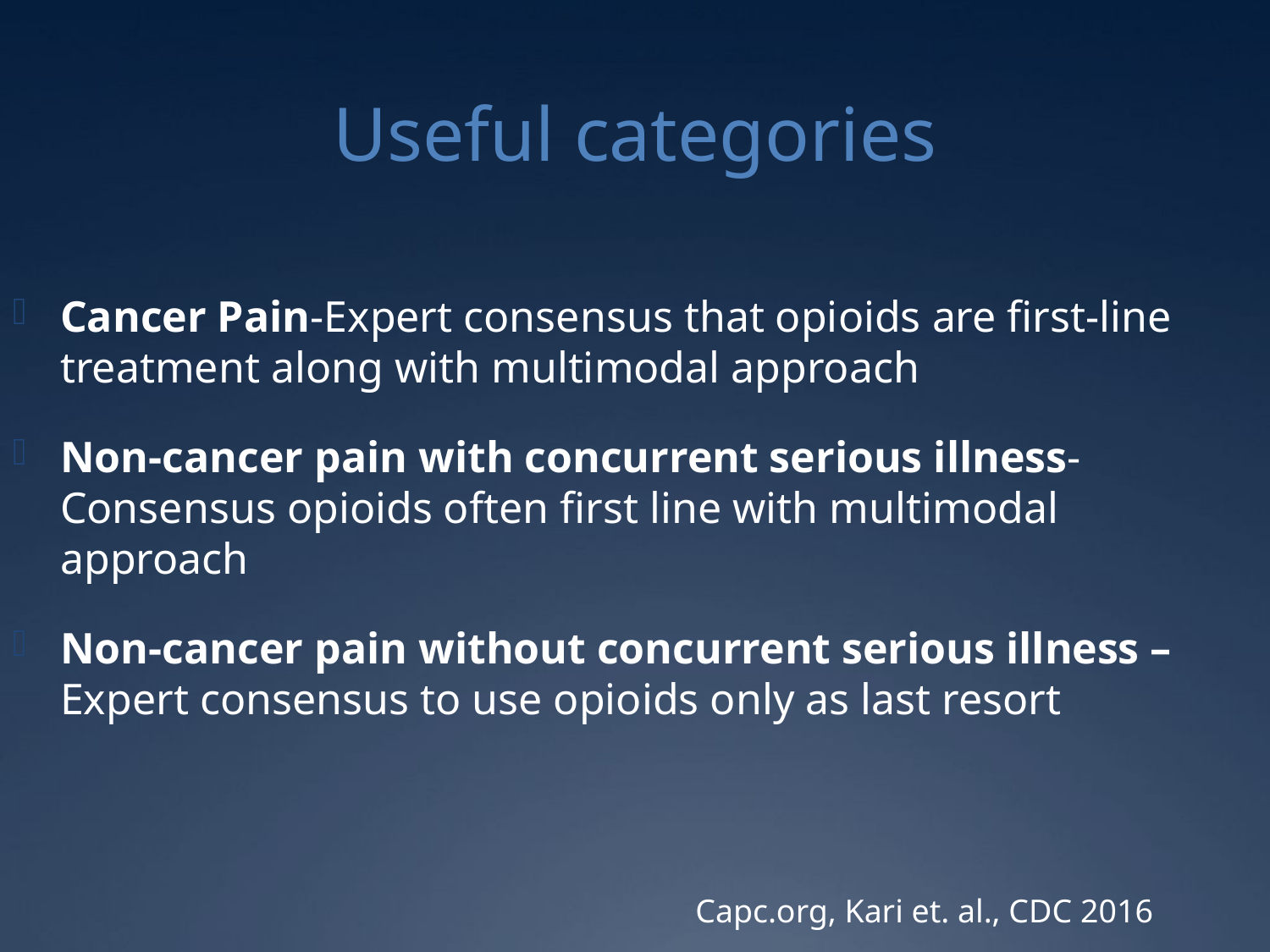

# Useful categories
Cancer Pain-Expert consensus that opioids are first-line treatment along with multimodal approach
Non-cancer pain with concurrent serious illness-Consensus opioids often first line with multimodal approach
Non-cancer pain without concurrent serious illness –Expert consensus to use opioids only as last resort
Capc.org, Kari et. al., CDC 2016

## Slide 11
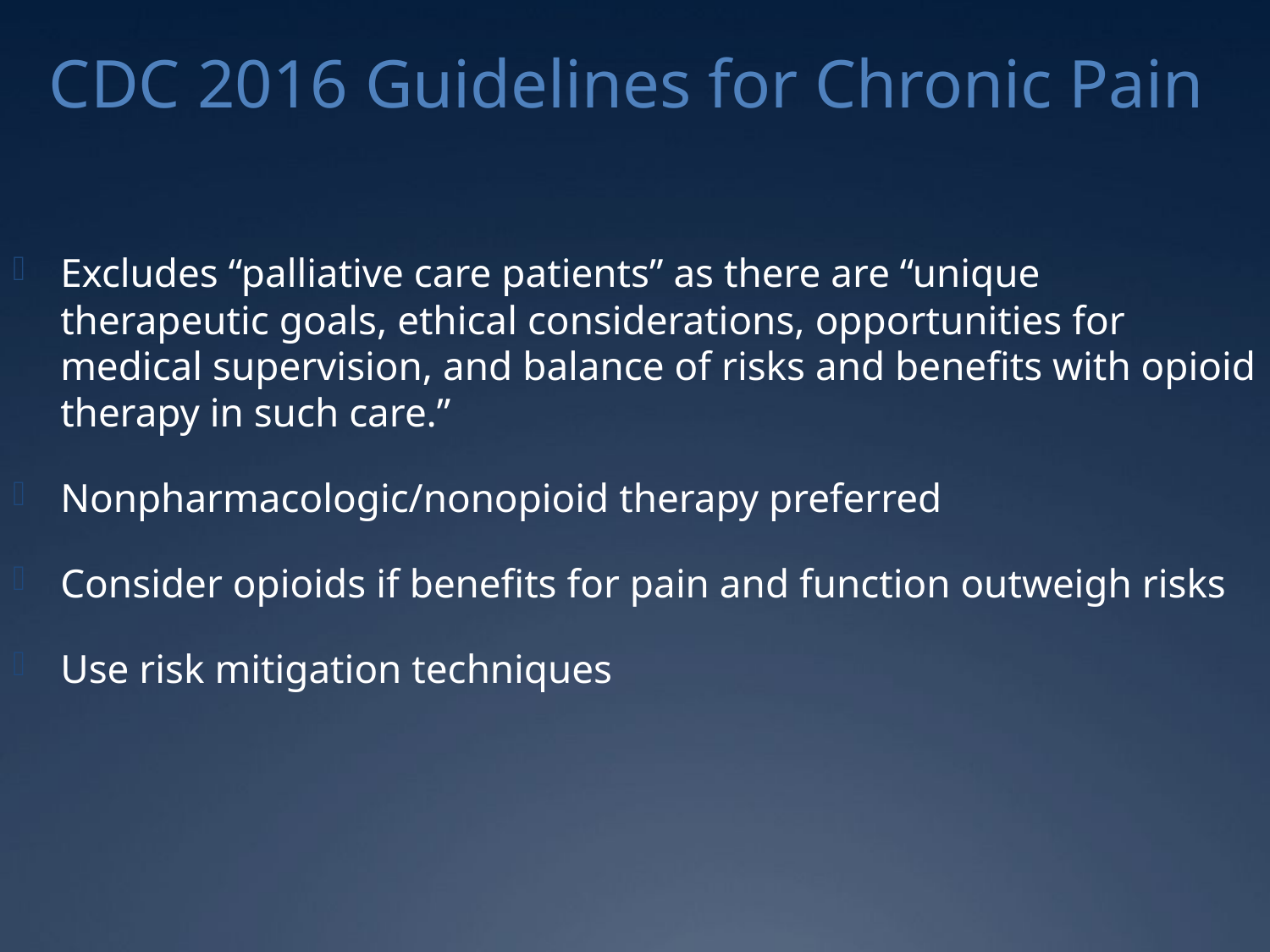

# CDC 2016 Guidelines for Chronic Pain
Excludes “palliative care patients” as there are “unique therapeutic goals, ethical considerations, opportunities for medical supervision, and balance of risks and benefits with opioid therapy in such care.”
Nonpharmacologic/nonopioid therapy preferred
Consider opioids if benefits for pain and function outweigh risks
Use risk mitigation techniques

## Slide 12
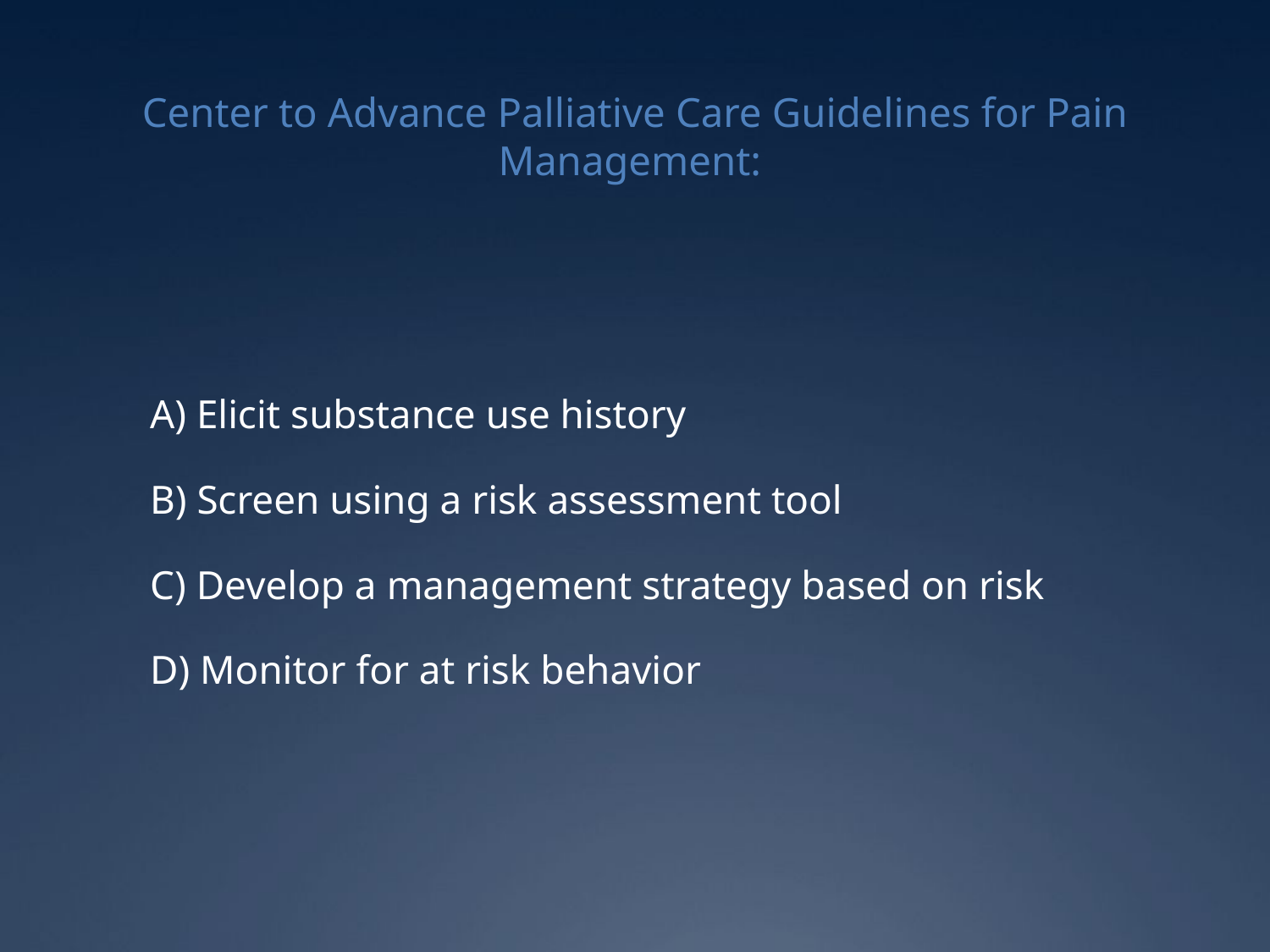

# Center to Advance Palliative Care Guidelines for Pain Management:
A) Elicit substance use history
B) Screen using a risk assessment tool
C) Develop a management strategy based on risk
D) Monitor for at risk behavior

## Slide 13
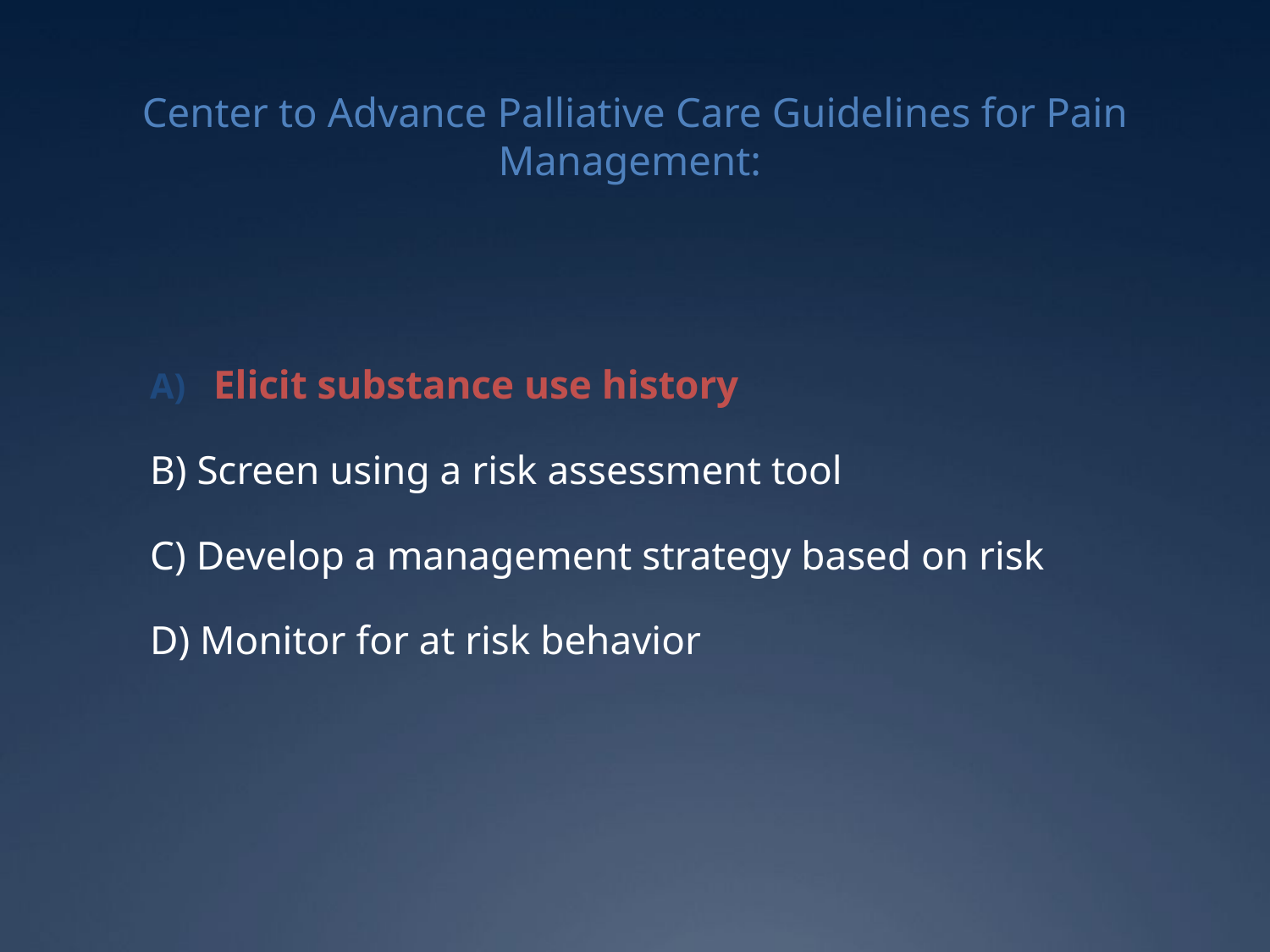

# Center to Advance Palliative Care Guidelines for Pain Management:
Elicit substance use history
B) Screen using a risk assessment tool
C) Develop a management strategy based on risk
D) Monitor for at risk behavior

## Slide 14
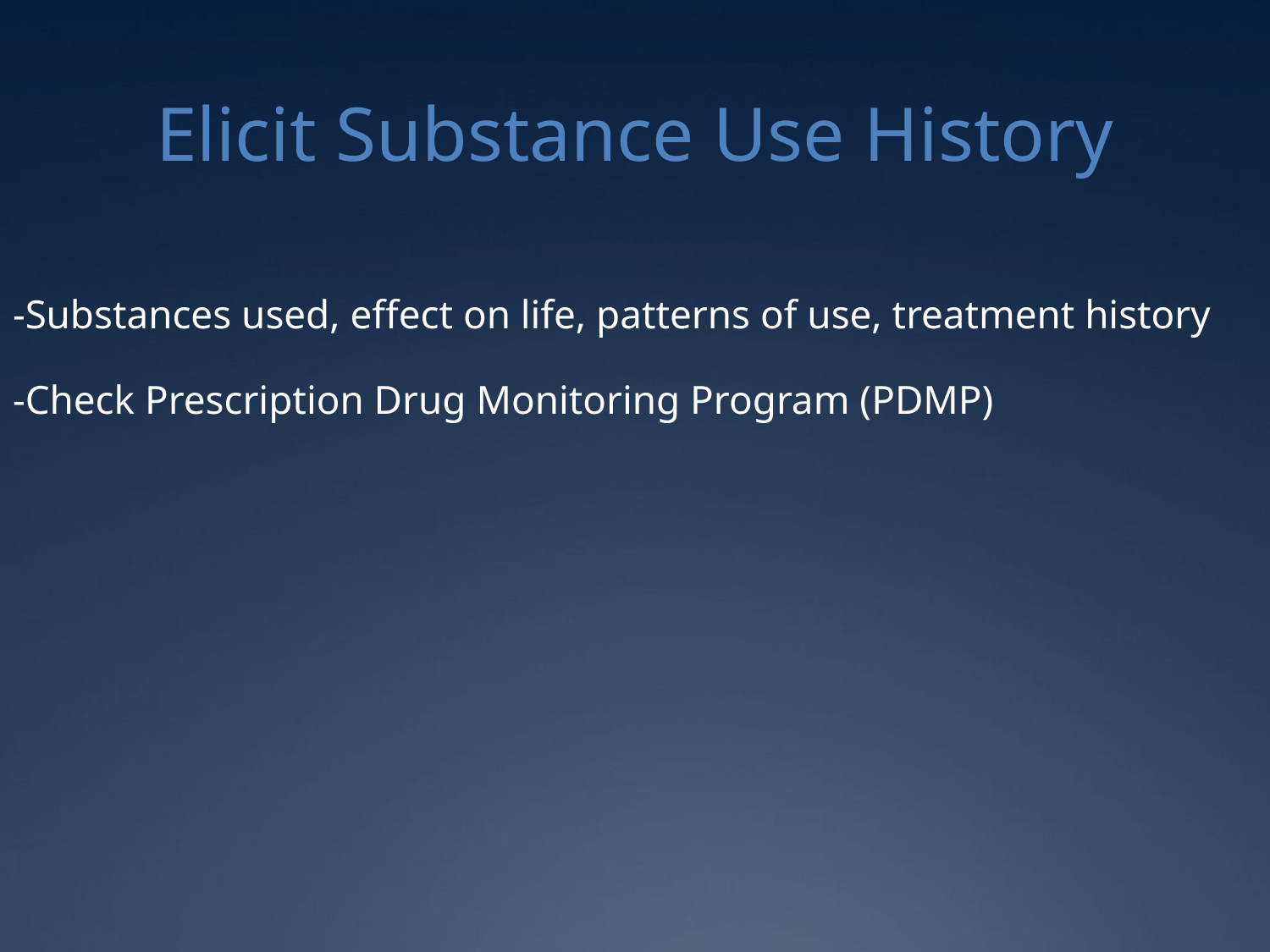

# Elicit Substance Use History
-Substances used, effect on life, patterns of use, treatment history
-Check Prescription Drug Monitoring Program (PDMP)

## Slide 15
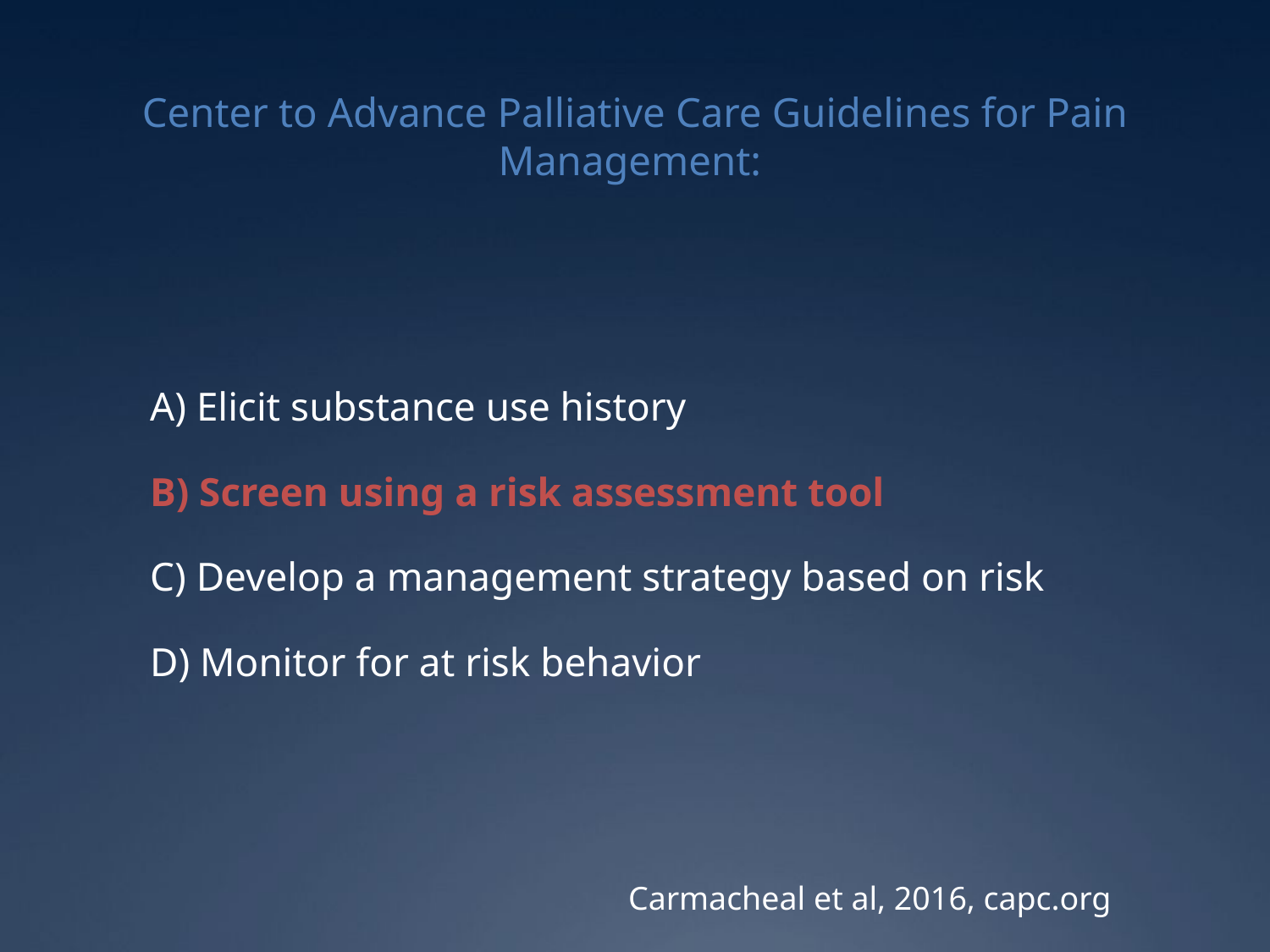

# Center to Advance Palliative Care Guidelines for Pain Management:
A) Elicit substance use history
B) Screen using a risk assessment tool
C) Develop a management strategy based on risk
D) Monitor for at risk behavior
Carmacheal et al, 2016, capc.org

## Slide 16
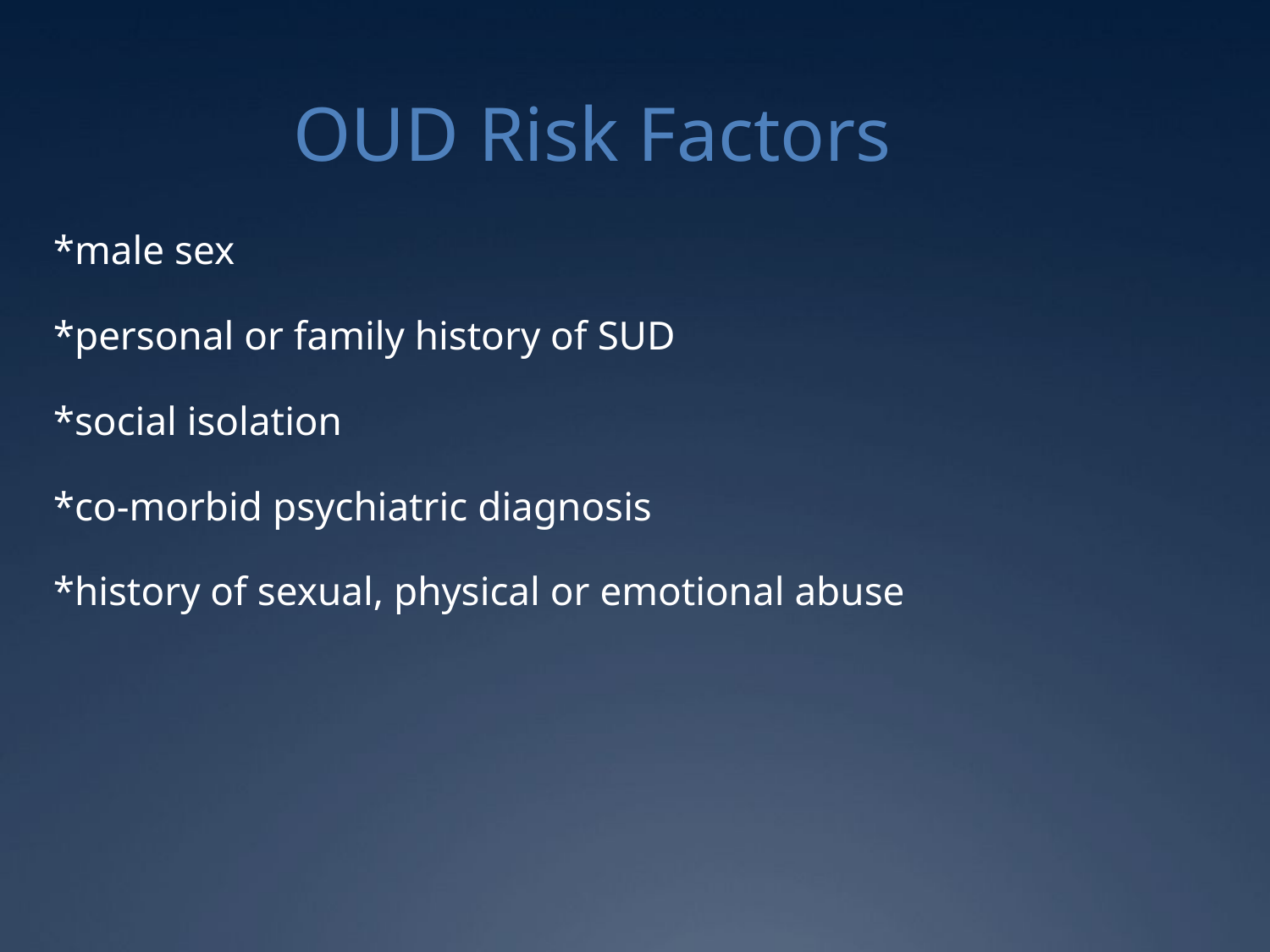

# OUD Risk Factors
 *male sex
 *personal or family history of SUD
 *social isolation
 *co-morbid psychiatric diagnosis
 *history of sexual, physical or emotional abuse

## Slide 17
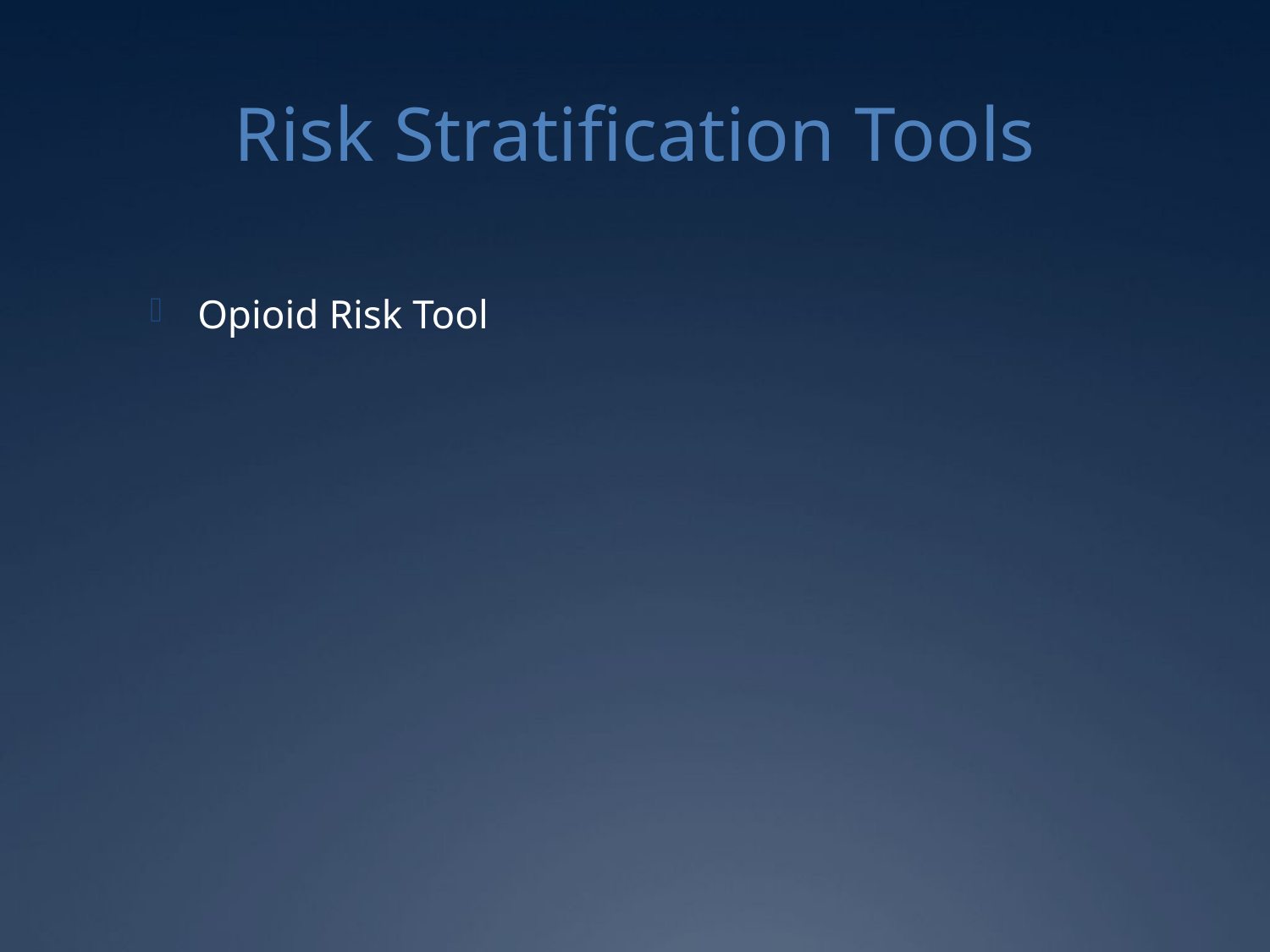

# Risk Stratification Tools
Opioid Risk Tool

## Slide 18
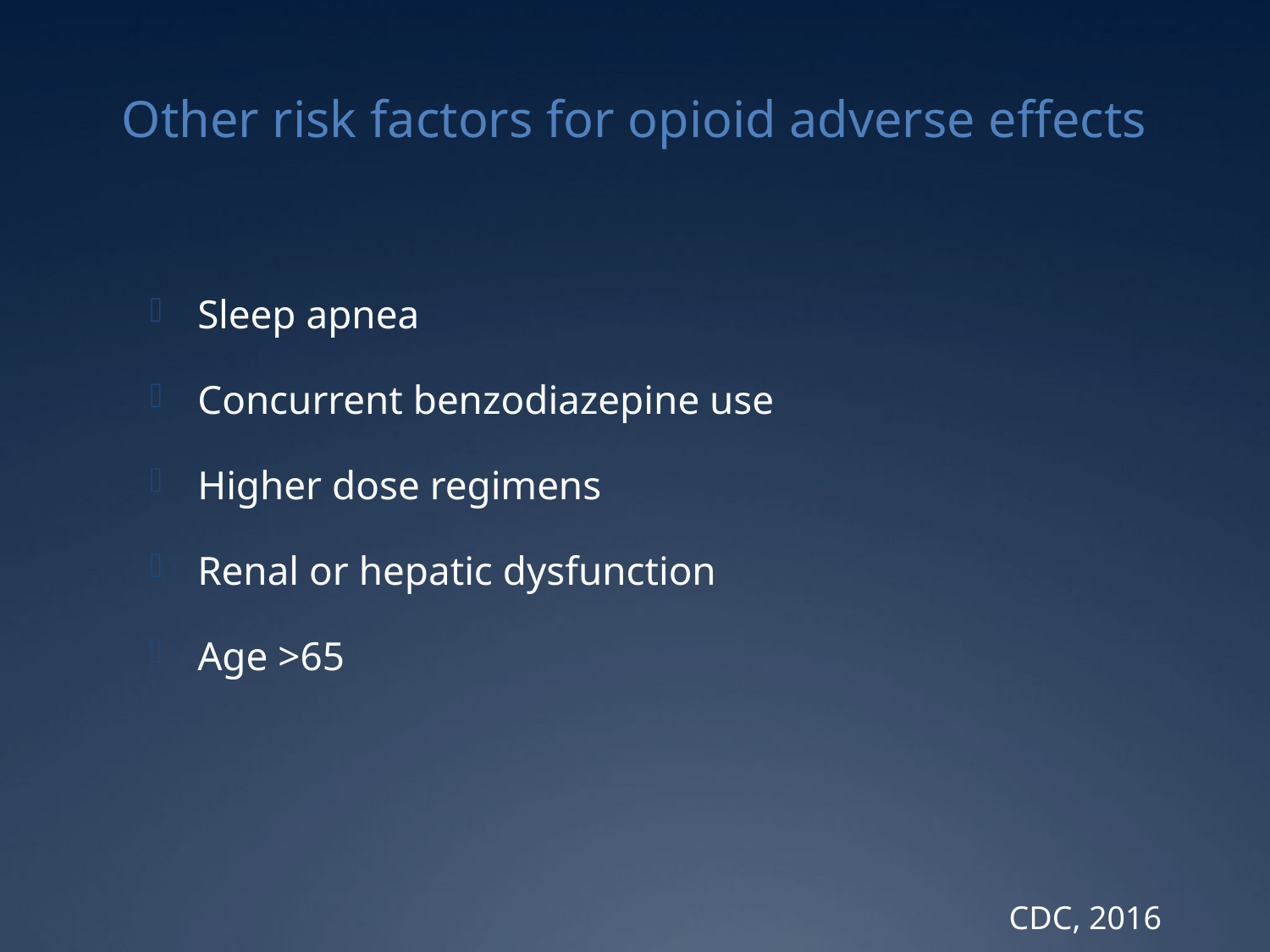

# Other risk factors for opioid adverse effects
Sleep apnea
Concurrent benzodiazepine use
Higher dose regimens
Renal or hepatic dysfunction
Age >65
CDC, 2016

## Slide 19
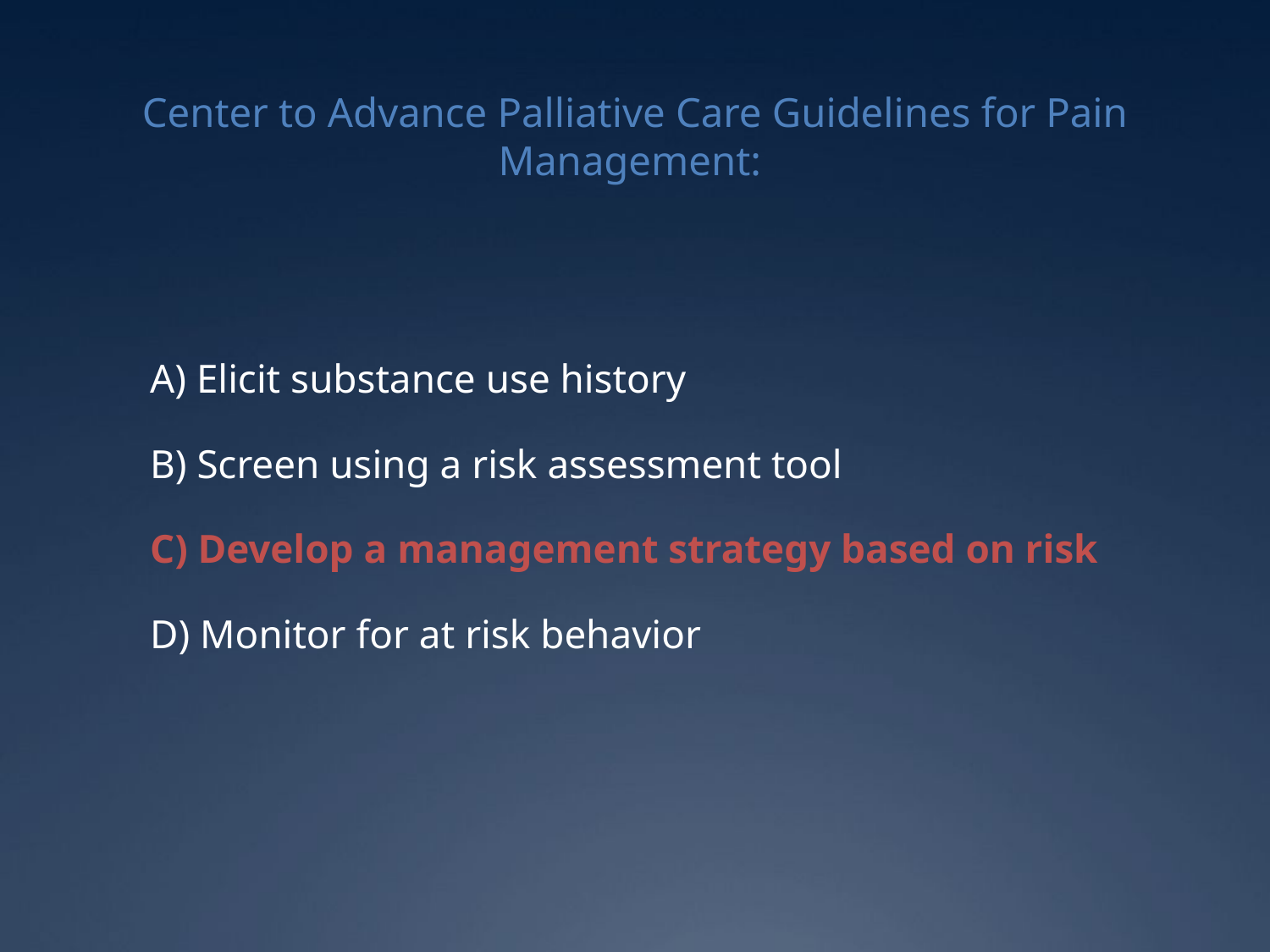

# Center to Advance Palliative Care Guidelines for Pain Management:
A) Elicit substance use history
B) Screen using a risk assessment tool
C) Develop a management strategy based on risk
D) Monitor for at risk behavior

## Slide 20
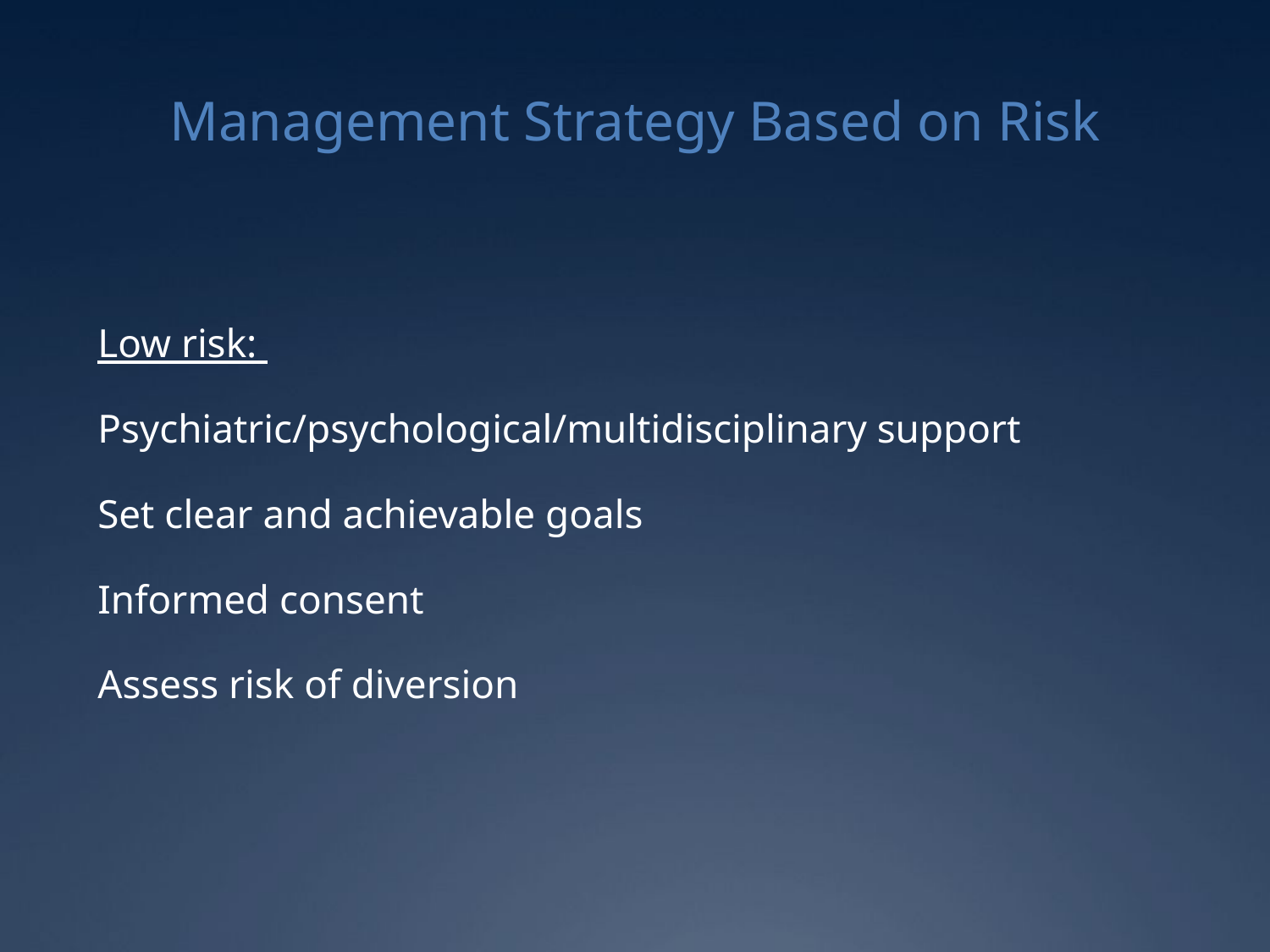

# Management Strategy Based on Risk
Low risk:
Psychiatric/psychological/multidisciplinary support
Set clear and achievable goals
Informed consent
Assess risk of diversion

## Slide 21
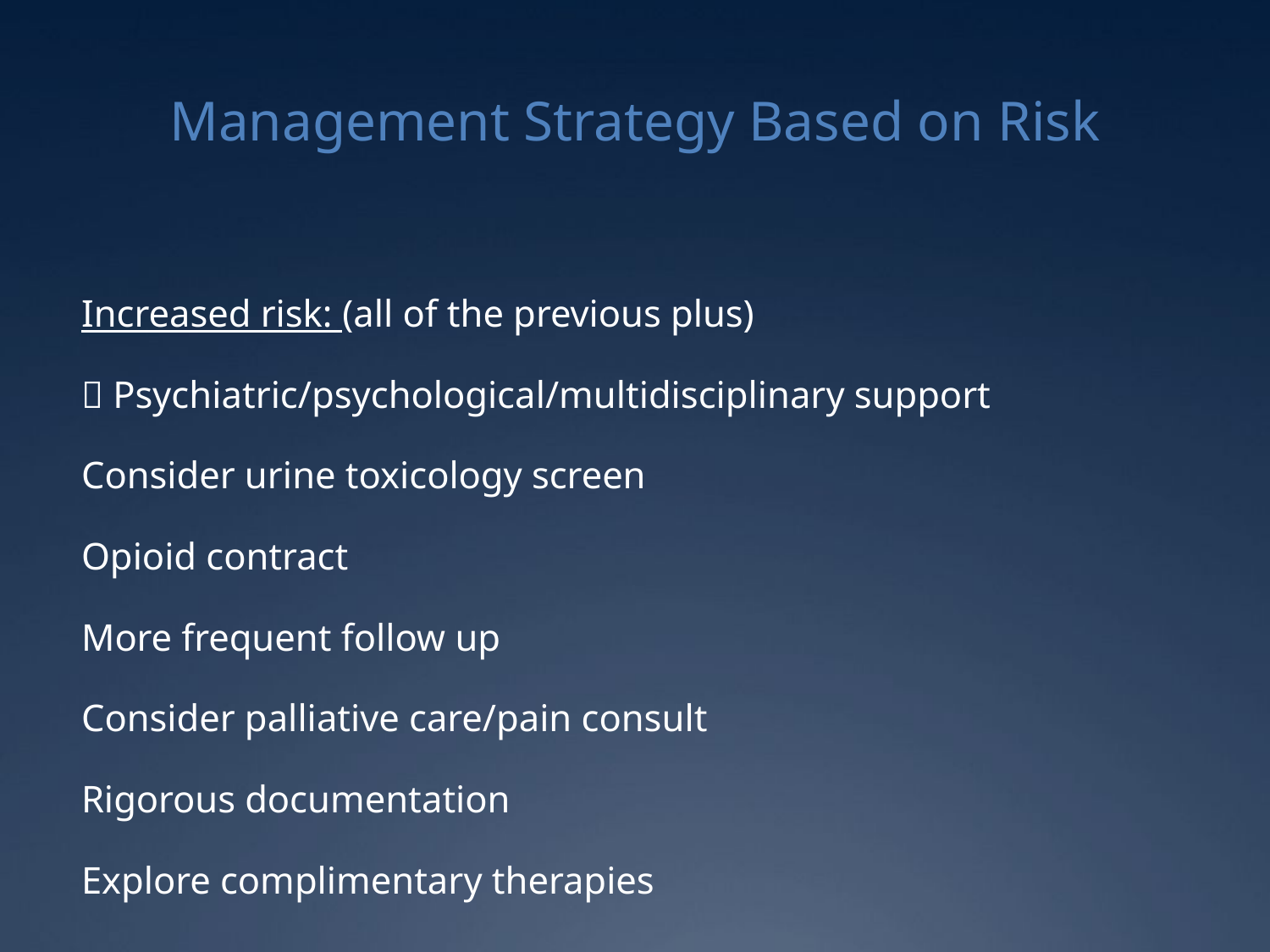

# Management Strategy Based on Risk
Increased risk: (all of the previous plus)
 Psychiatric/psychological/multidisciplinary support
Consider urine toxicology screen
Opioid contract
More frequent follow up
Consider palliative care/pain consult
Rigorous documentation
Explore complimentary therapies

## Slide 22
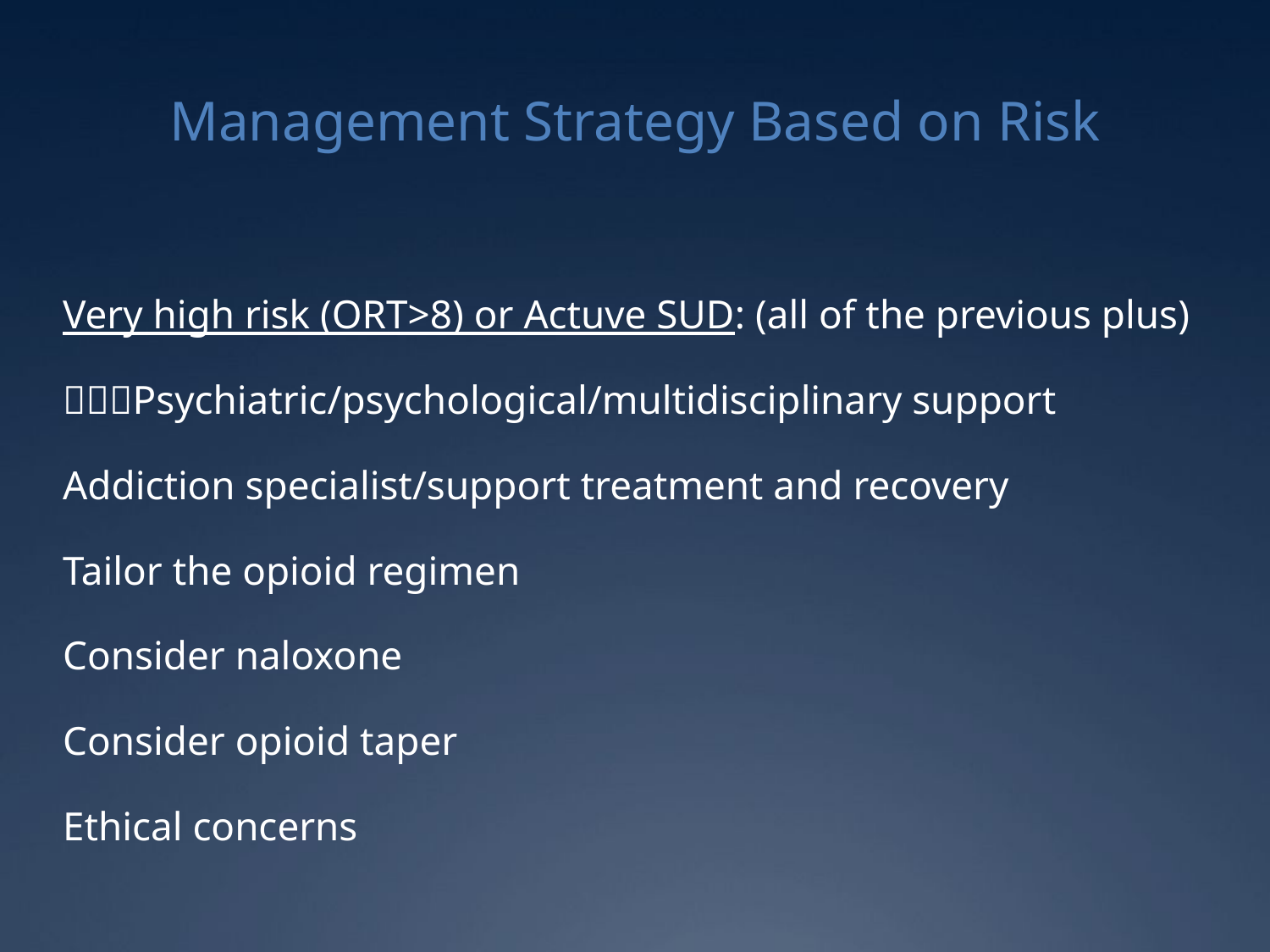

# Management Strategy Based on Risk
Very high risk (ORT>8) or Actuve SUD: (all of the previous plus)
Psychiatric/psychological/multidisciplinary support
Addiction specialist/support treatment and recovery
Tailor the opioid regimen
Consider naloxone
Consider opioid taper
Ethical concerns

## Slide 23
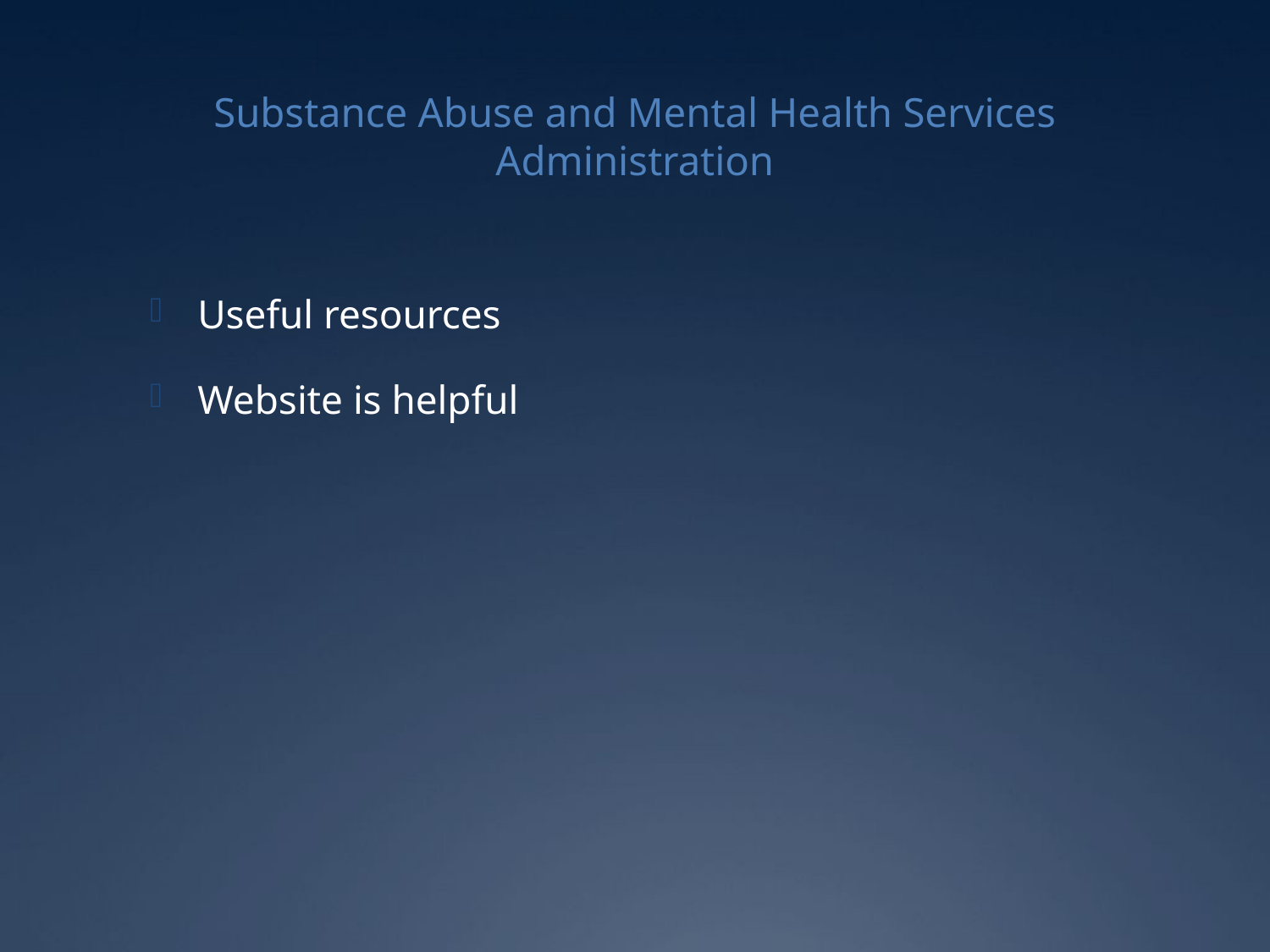

# Substance Abuse and Mental Health Services Administration
Useful resources
Website is helpful

## Slide 24
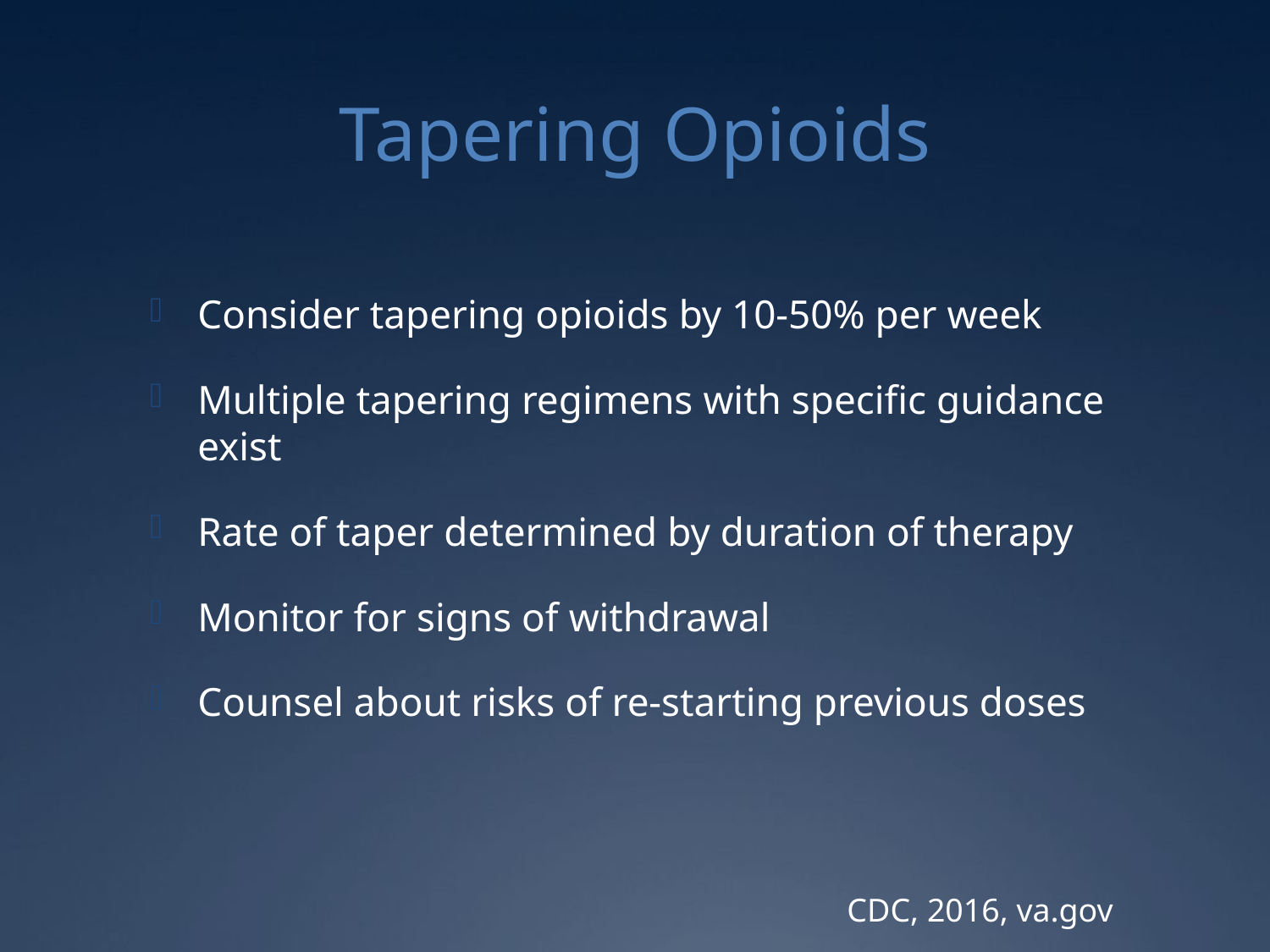

# Tapering Opioids
Consider tapering opioids by 10-50% per week
Multiple tapering regimens with specific guidance exist
Rate of taper determined by duration of therapy
Monitor for signs of withdrawal
Counsel about risks of re-starting previous doses
CDC, 2016, va.gov

## Slide 25
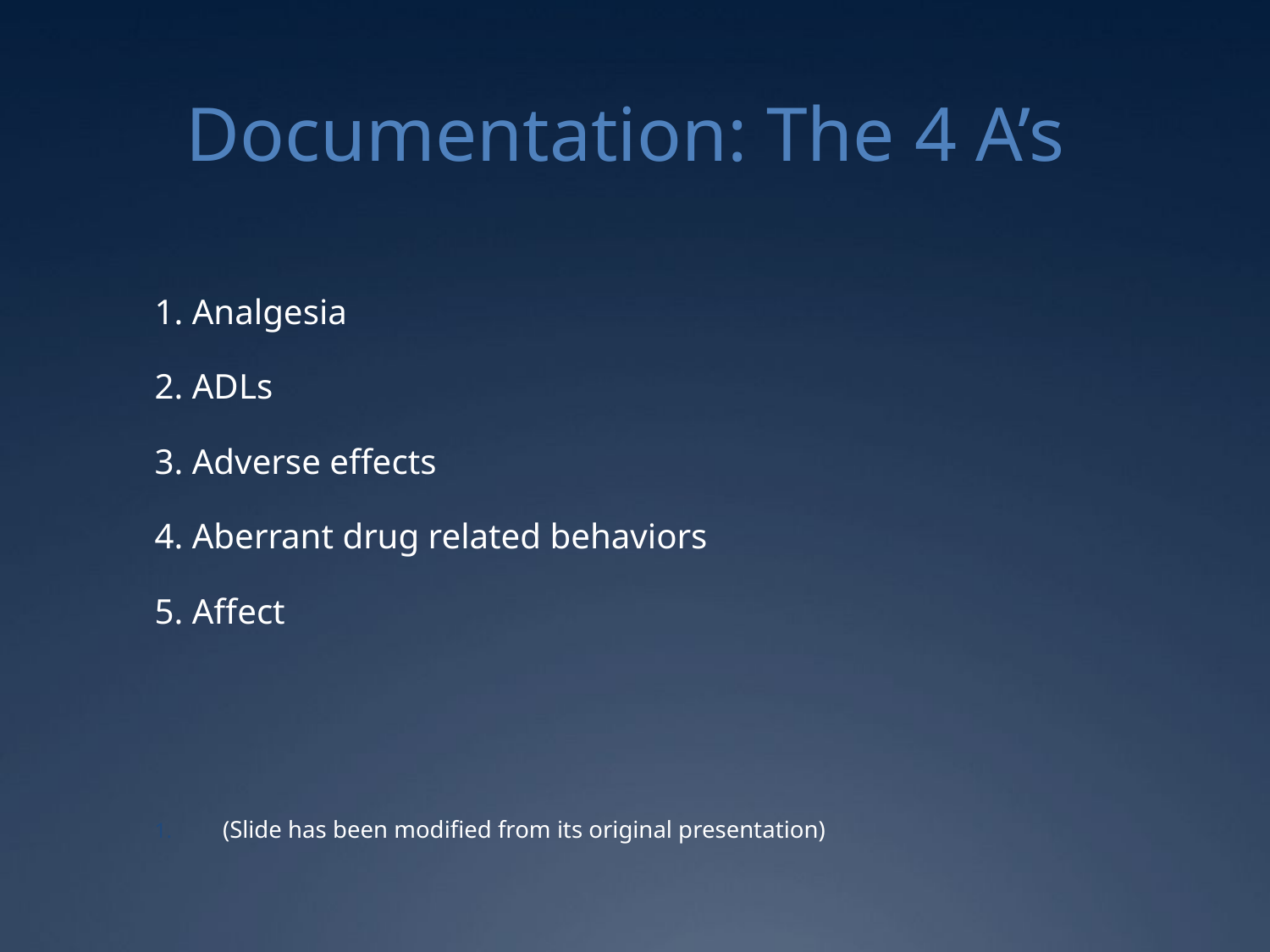

# Documentation: The 4 A’s
1. Analgesia
2. ADLs
3. Adverse effects
4. Aberrant drug related behaviors
5. Affect
(Slide has been modified from its original presentation)

## Slide 26
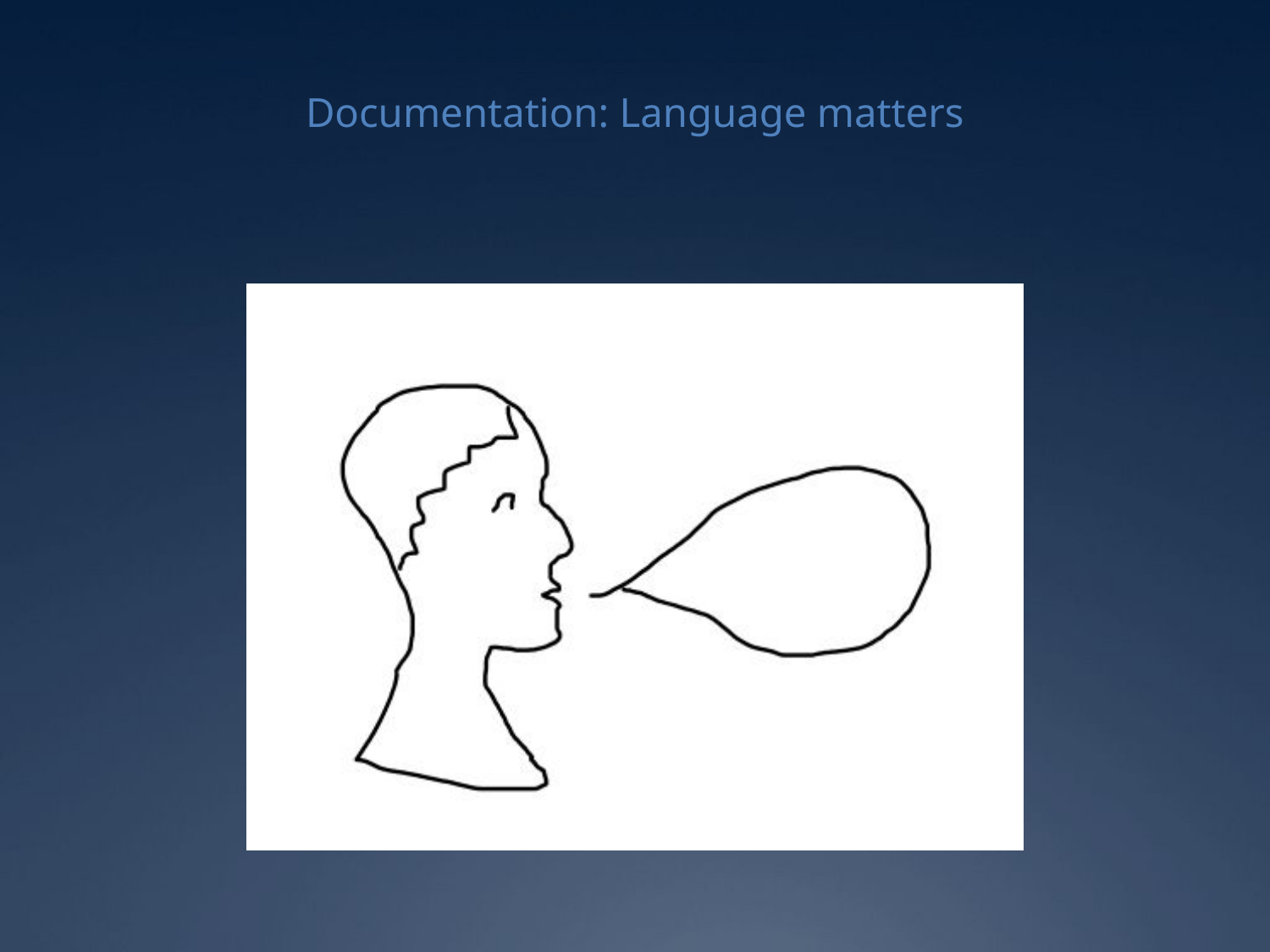

# Documentation: Language matters

## Slide 27
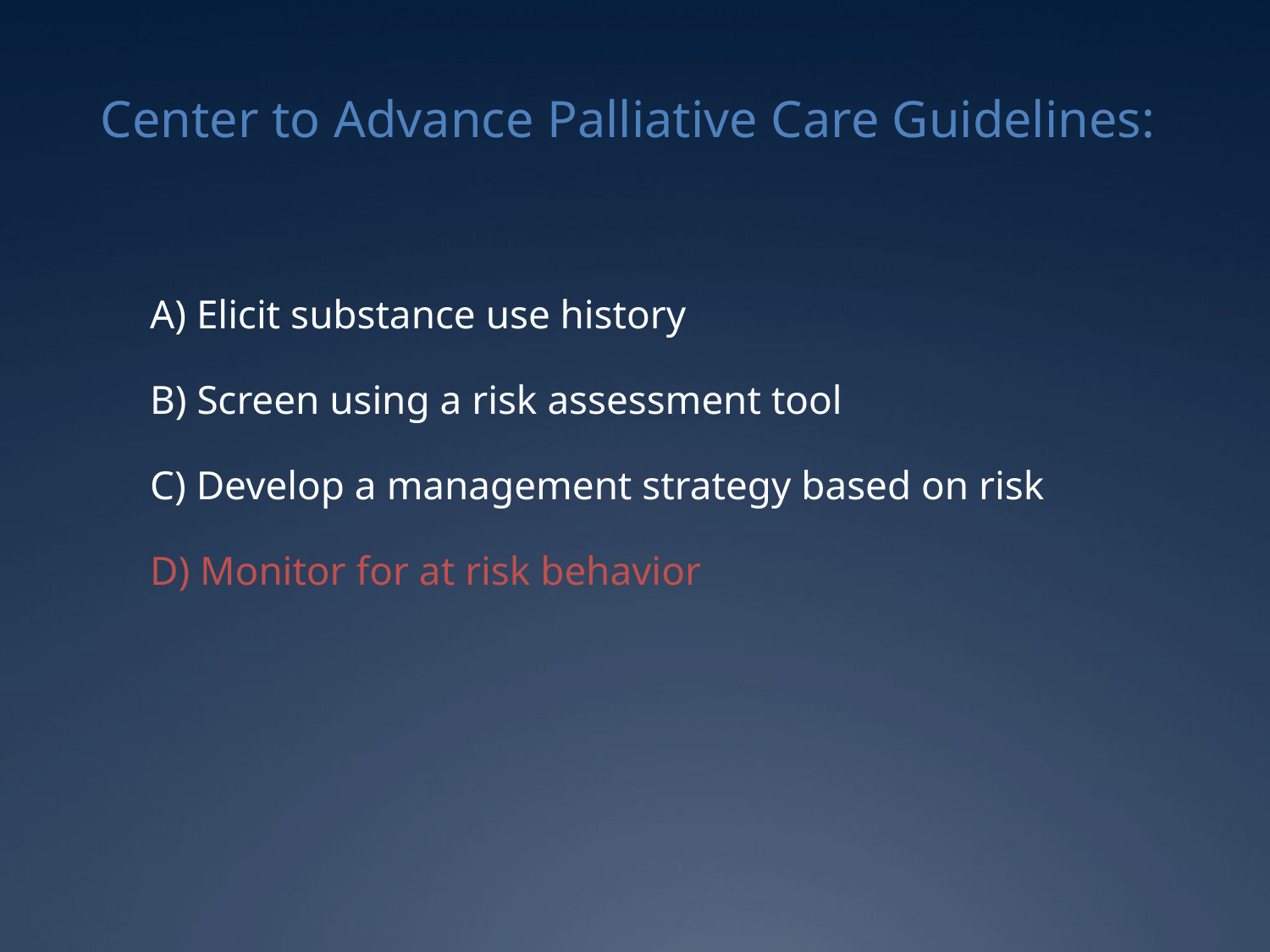

# Center to Advance Palliative Care Guidelines:
A) Elicit substance use history
B) Screen using a risk assessment tool
C) Develop a management strategy based on risk
D) Monitor for at risk behavior

## Slide 28
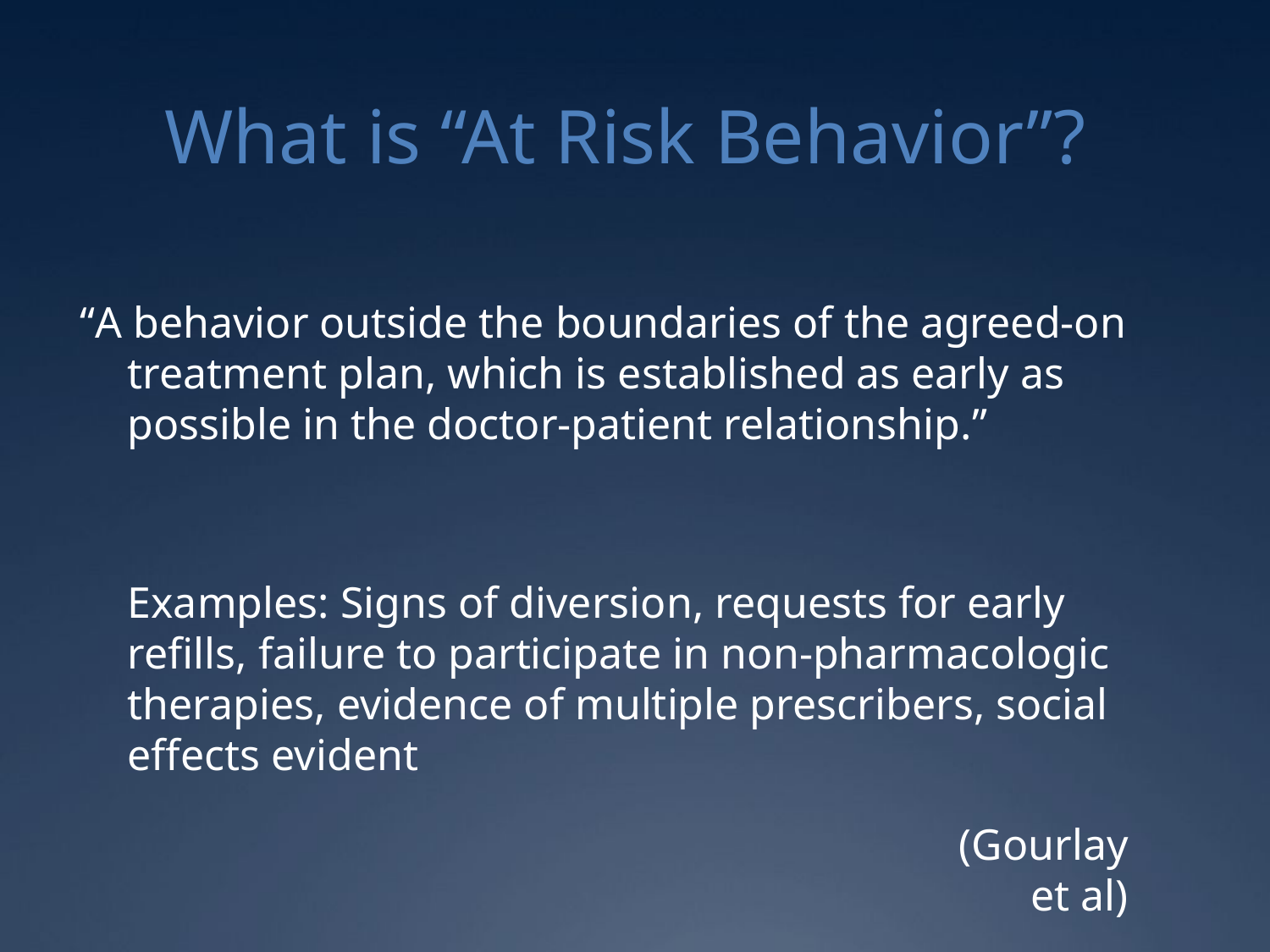

# What is “At Risk Behavior”?
“A behavior outside the boundaries of the agreed-on treatment plan, which is established as early as possible in the doctor-patient relationship.”
	Examples: Signs of diversion, requests for early refills, failure to participate in non-pharmacologic therapies, evidence of multiple prescribers, social effects evident
 (Gourlay et al)

## Slide 29
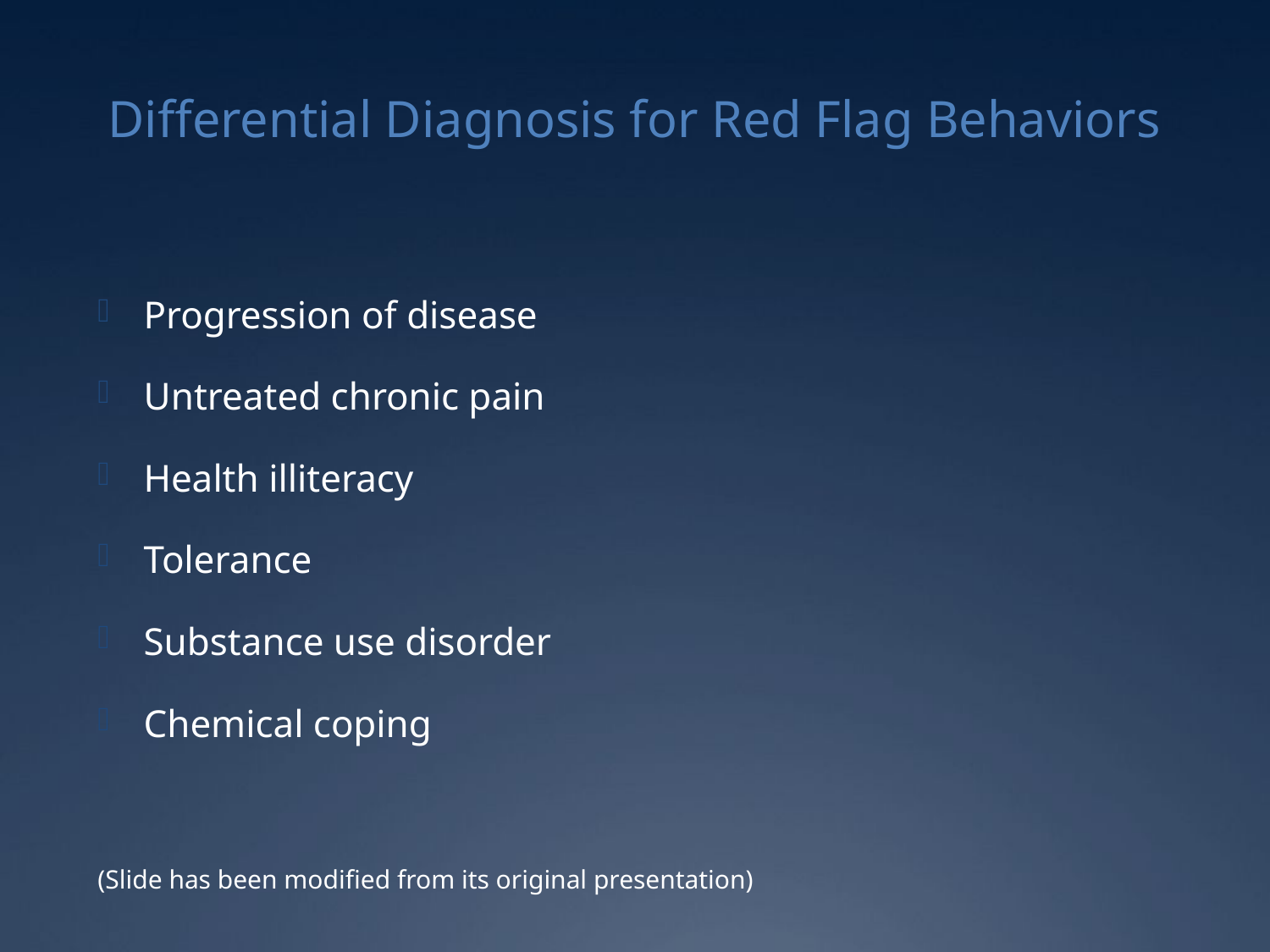

# Differential Diagnosis for Red Flag Behaviors
Progression of disease
Untreated chronic pain
Health illiteracy
Tolerance
Substance use disorder
Chemical coping
(Slide has been modified from its original presentation)

## Slide 30
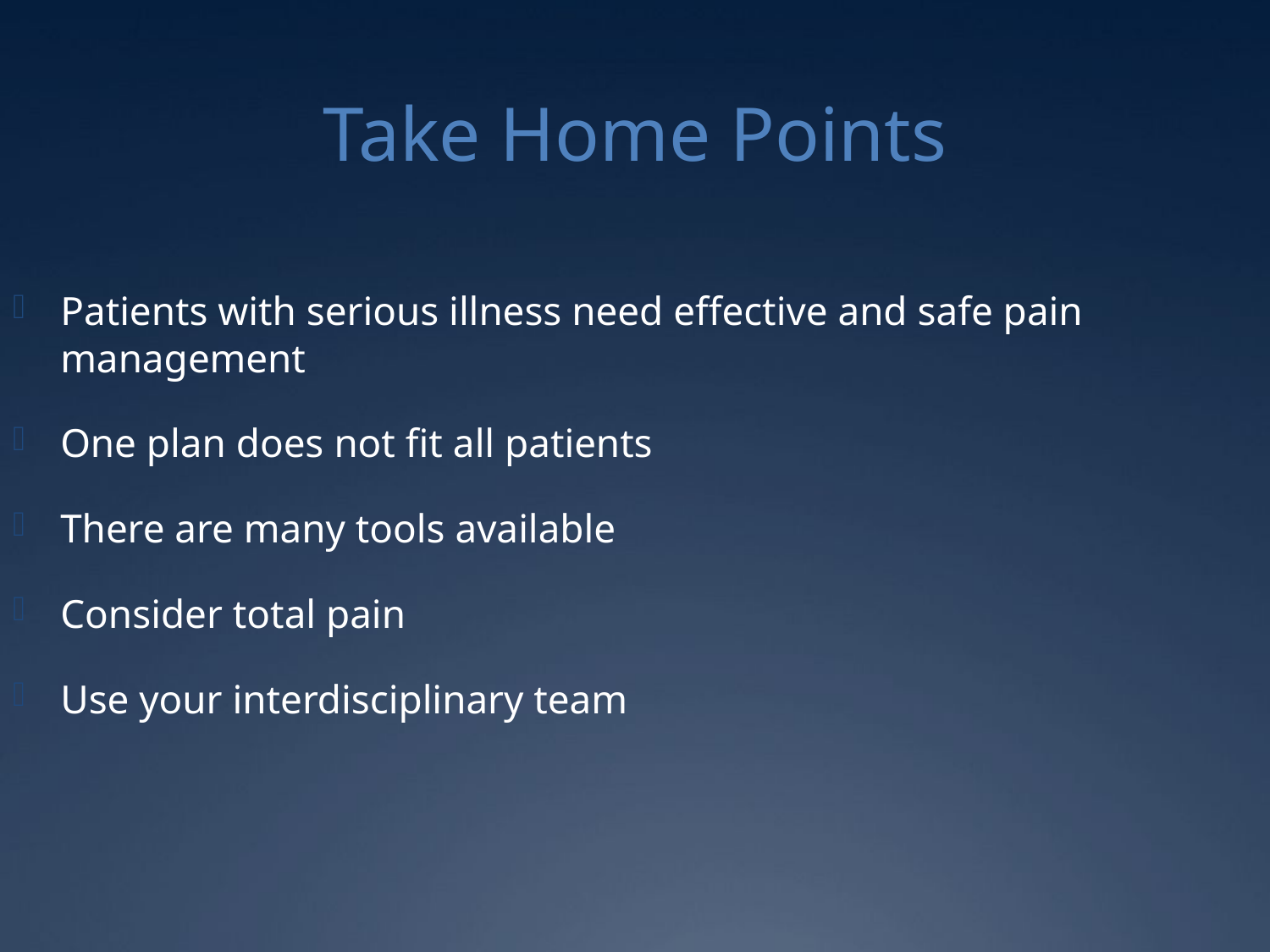

# Take Home Points
Patients with serious illness need effective and safe pain management
One plan does not fit all patients
There are many tools available
Consider total pain
Use your interdisciplinary team

## Slide 31
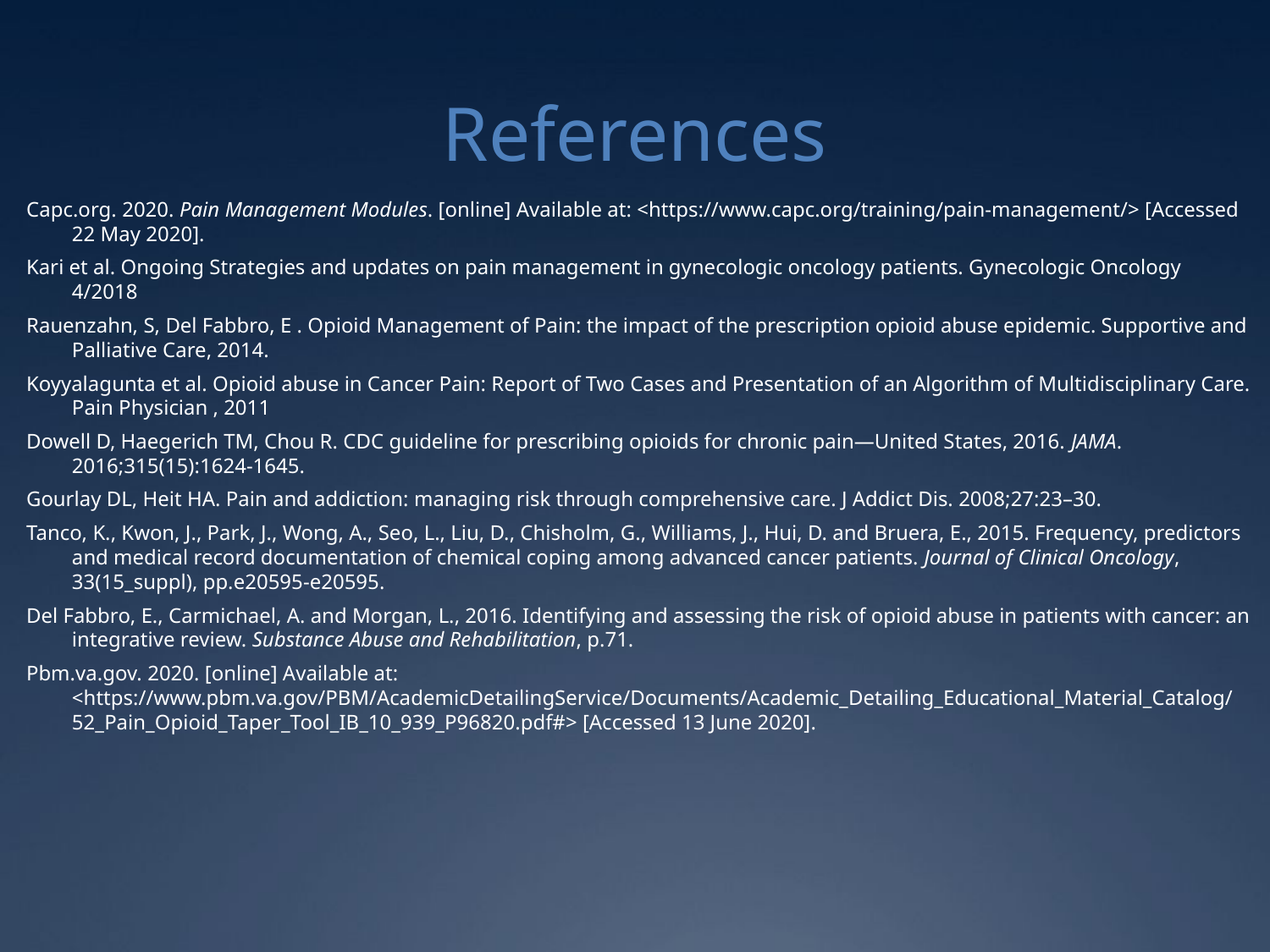

# References
Capc.org. 2020. Pain Management Modules. [online] Available at: <https://www.capc.org/training/pain-management/> [Accessed 22 May 2020].
Kari et al. Ongoing Strategies and updates on pain management in gynecologic oncology patients. Gynecologic Oncology 4/2018
Rauenzahn, S, Del Fabbro, E . Opioid Management of Pain: the impact of the prescription opioid abuse epidemic. Supportive and Palliative Care, 2014.
Koyyalagunta et al. Opioid abuse in Cancer Pain: Report of Two Cases and Presentation of an Algorithm of Multidisciplinary Care. Pain Physician , 2011
Dowell D, Haegerich TM, Chou R. CDC guideline for prescribing opioids for chronic pain—United States, 2016. JAMA. 2016;315(15):1624-1645.
Gourlay DL, Heit HA. Pain and addiction: managing risk through comprehensive care. J Addict Dis. 2008;27:23–30.
Tanco, K., Kwon, J., Park, J., Wong, A., Seo, L., Liu, D., Chisholm, G., Williams, J., Hui, D. and Bruera, E., 2015. Frequency, predictors and medical record documentation of chemical coping among advanced cancer patients. Journal of Clinical Oncology, 33(15_suppl), pp.e20595-e20595.
Del Fabbro, E., Carmichael, A. and Morgan, L., 2016. Identifying and assessing the risk of opioid abuse in patients with cancer: an integrative review. Substance Abuse and Rehabilitation, p.71.
Pbm.va.gov. 2020. [online] Available at: <https://www.pbm.va.gov/PBM/AcademicDetailingService/Documents/Academic_Detailing_Educational_Material_Catalog/52_Pain_Opioid_Taper_Tool_IB_10_939_P96820.pdf#> [Accessed 13 June 2020].
