# Supplementary material for: Teaching Pain Management in Serious Illness in the Era of the Opioid Epidemic: A Team-Based Intervention
Source: MedEdPORTAL. 2020 Oct 30;16:11006. doi: 10.15766/mep_2374-8265.11006 (PMC7597940; doi:10.15766/mep_2374-8265.11006)
Supplement: Supplementary file 1 — Case.docxPain Management & Risk Presentation.pptxPain & Risk Survey.docx [file mep_2374-8265.11006-s001.zip › C. Pain & Risk Survey.docx]

**Survey on Attitudes around Opioid Prescribing in Serious Illness**

|  | Strongly Disagree | Disagree | Neutral | Agree | Strongly Agree |
| --- | --- | --- | --- | --- | --- |
| 1. I am familiar with the concept of total pain. |  |  |  |  |  |
| 2. Opioids can be a useful tool in pain management for patients with serious illness. |  |  |  |  |  |
| 3. It is important for me to learn how to prescribe opioids for patients with serious illness. |  |  |  |  |  |
| 4. A patient with an opioid use disorder and pain due to serious illness should not be prescribed opioids. |  |  |  |  |  |
| 5. I am familiar with some strategies to help guide me when caring for patients with high risk of substance use disorder and pain in the setting of serious illness. |  |  |  |  |  |
| 6. I am familiar with how to safely taper an opioid regimen when appropriate. |  |  |  |  |  |
| 7. When a patient taking an opioid demonstrates “red flag” signs it means they have an opioid use disorder. |  |  |  |  |  |
| 8. A team approach is helpful when treating patients with serious illness at risk of opioid use disorder. |  |  |  |  |  |
| 9. I am familiar with how an interdisciplinary team might approach care of a patient with serious illness at risk for opioid use disorder. |  |  |  |  |  |
| 10. I am familiar with how a palliative care pharmacist functions as part of the interdisciplinary team. |  |  |  |  |  |
| 11. I am familiar with how a palliative care social worker functions as part of the interdisciplinary team. |  |  |  |  |  |
| 12. I am familiar with how a palliative care spiritual care provider functions as part of the interdisciplinary team. |  |  |  |  |  |
| 13. I am familiar with how a palliative care physician functions as part of the interdisciplinary team. |  |  |  |  |  |

**Directions:**

Indicate the extent to which you agree or disagree with the statements by checking the corresponding box.

**Was this session helpful? Why or why not?**
